# A Galactose-Functionalized Pyrrolopyrrole *Aza*-BODIPY for Highly Efficient Detection of Eight Aliphatic and Aromatic Biogenic Amines: Monitoring Food Freshness and Bioimaging

**DOI:** 10.3390/bios15080542

**Published:** 2025-08-18

**Authors:** Yujing Gan, Bingli Lu, Jintian Zhong, Xueguagn Ran, Derong Cao, Lingyun Wang

**Affiliations:** 1 School of Chemistry and Chemical Engineering, South China University of Technology, 381 Wushan Road, Guangzhou 510640, China; 2 The State Key Laboratory of Swine and Poultry Breeding Industry, Key Laboratory of Animal Nutrition and Feed Science in South China, Ministry of Agriculture and Rural Affairs, Guangdong Provincial Key Laboratory of Animal Breeding and Nutrition, Institute of Animal Science, Guangdong Academy of Agricultural Sciences, Guangzhou 510640, China

**Keywords:** aromatic biogenic amines, chromophore reaction, galactose, synthesis, noncovalent interactions, aliphatic biogenic amines

## Abstract

The detection of aliphatic and aromatic biogenic amines (BAs) is important in food spoilage, environmental monitoring, and disease diagnosis and treatment. Existing fluorescent probes predominantly detect aliphatic BAs with single signal variation and low sensitivity, impairing the adaptability of discriminative sensing platforms. Herein, we present a visual chemosensor (galactose-functionalized pyrrolopyrrole *aza*-BODIPY, **PPAB-Gal**) that simultaneously detects eight aliphatic and aromatic BAs in a real-time and intuitive way based on their unique electronic and structural features. Our findings reveal that the dual colorimetric and ratiometric emission changes are rapidly produced in presence of eight BAs through a noncovalent interaction (π–π stacking and hydrogen bond)-assisted chromophore reaction. Specifically, other lone-pair electrons containing compounds, such as secondary amines, tertiary amines, NH_3_, and thiol, fail to exhibit these changes. As a result, superior sensing performances with distinctly dual signals (Δλ_ab_ = 130 nm, Δλ_em_ = 150 nm), a low LOD (~25 nM), and fast response time (<2 min) were obtained. Based on these advantages, a qualitative and smartphone-assisted sensing platform with a **PPAB-Gal**-loaded TLC plate is developed for visual detection of putrescine and cadaverine vapor. More importantly, we construct a connection between a standard quantitative index for the TVBN value and fluorescence signals to quantitatively determine the freshness of tuna and shrimp, and the method is facile and convenient for real-time and on-site detection in practical application. Furthermore, since the overexpressed spermine is an important biomarker of cancer diagnosis and treatment, **PPAB-Gal** NPs can be used to ratiometrically image spermine in living cells. This work provides a promising sensing method for BAs with a novel fluorescent material in food safety fields and biomedical assays.

## 1. Introduction

The important food sources in our lives, such as cheese, fish, shrimp, meat, or dairy products, are rich in proteins, lipids and vitamins [1,2]. Nevertheless, they are easily spoiled and generate biogenic amines (BAs), where proteins are decomposed by endogenous enzymes and pathogenic microorganisms [3]. Both typical aliphatic BAs (spermine, spermidine, putrescine, and cadaverine) and aromatic BAs (tyramine, histamine, 2-phenylethylamine, and tryptamine) are released. Excessive amounts of BAs are toxic and foul-smelling, posing a serious threat to the environment and public health. In particular, high levels of histamine in seafood are responsible for scombroid poisoning [4,5,6]. Excessive concentrations of 2-phenylethylamine induce high blood pressure, neurotoxicity, and allergies [7]. Overdoses of tryptamine cause hallucinations, shock, or sudden death. Meanwhile, the inhibitory effects of putrescine and cadaverine on histamine-related enzymes have been demonstrated [8], worsening the painful sensation. Among them, tyramine and histamine are the most common intoxication sources. The greatest risk from BAs derives from their accumulation in certain foods [9,10,11,12,13]. The guidance levels for histamine, tryptamine, and 2-phenylethylamine are 50, 100–800, and 30 mg/kg, respectively, in aquatic products as established by the FDA [14,15,16]. On the other hand, spermine serves as an essential modulator of multiple intracellular processes, including cellular proliferation, progression, viability, protein synthesis, and gene expression [17]. The high level of spermine is associated with cancer as well as gastrointestinal and immunological diseases. Therefore, developing sensing materials capable of simultaneous detecting of aliphatic and aromatic BAs is vital and urgent for human health and food quality.

Traditional BA analysis methods include HPLC, LC–MS, GC–MS, ELISAs, sensor arrays, electrophoresis, and electronic nose, etc. They can accurately determinate BA contents; however, some drawbacks such as time-consuming and complex operation procedures, complicated and expensive instruments, and the requirement of professional personnel are encountered. It is noteworthy that chemical reaction-based fluorescent probes for BA detection have remarkable advantages due to the high selectivity, fast response, simple operation, and excellent sensitivity. Several sensing mechanisms including nucleophilic addition, chromophore reaction, *aza*-Michael addition, condensation reaction, formation of Schiff bases, and ester aminolysis reaction have been developed [18,19,20,21,22,23,24]. Unfortunately, these fluorescent probes just work on aliphatic BAs and fail to respond to aromatic BAs due to their large steric hindrance of the aromatic group and reduced nucleophilicity of the amine group [25,26]. At present, the detection of aromatic BAs mainly relies on instrumental methods, fluorescent covalent organic frameworks, metal organic frameworks, and fluorescent quantum dots based on acid-base or host-guest interactions [27,28,29,30]. Therefore, several issues are urgently needed to be solved in the field of BA detection. (1) The majority of chemical reaction-based fluorescent probes for aliphatic BA detection still have shortcomings, such as a sluggish reaction rate, a pure organic solvent detection system, strong background fluorescence, and inferior sensitivity. (2) Developing a new strategy to achieve aromatic BA detection is required. It remains extremely challenging to exploit a chemical reaction-based fluorescent probe that can simultaneously detect aliphatic and aromatic BAs. 

**Scheme 1:**
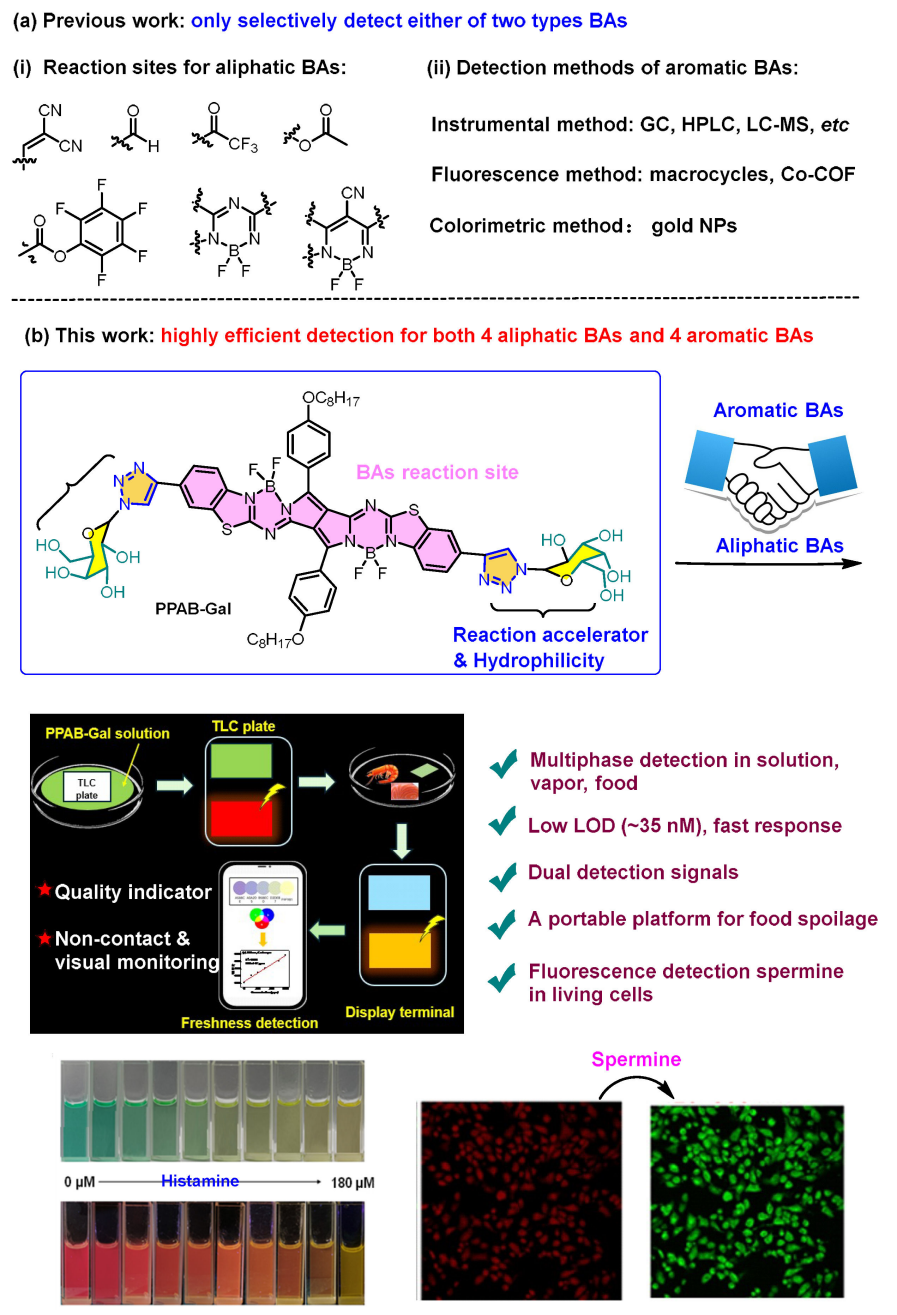
(**a**) Previous work for BAs sensing. (**b**) The design strategy of fluorescent probe (**PPAB-Gal**) in this work for aliphatic and aromatic BAs detection.

The largely conjugated pyrrolopyrrole *aza*-BODIPY (PPAB) fluorophore has intense absorption and fluorescence in the NIR region. Our group is the first to use PPAB to detect aliphatic BAs through a chromophore reaction mechanism [31,32,33,34]. With this objective and combined with our research interest, we constructed a fluorescent probe **PPAB-Gal** using PPAB as a fluorophore and recognition site linked to 1,2,3-triazole and a galactose group as the reaction accelerator (Scheme 1). The noncovalent interactions (hydrogen bond and π–π interaction, etc.) between **PPAB-Gal** and aliphatic/aromatic BAs would bring BAs to the proximity of the **PPAB-Gal**. As a result, subsequent intramolecular chromophore reaction between them would be highly accelerated, yielding superior sensing performance for aliphatic BAs and simultaneously detecting aromatic BAs with excellent sensitivity. Several advantages have been shown as follows. (1) Simultaneous detection of 8 BAs using a single reaction-based fluorescent probe in 20% DMSO /H_2_O (4/1, *v*/*v*) was achieved for the first time. (2) The superior sensing performance such as distinctly dual signals (Δλ_ab_ = 130 nm, Δλ_em_ = 150 nm) with ratiometric fluorescence for naked eye detection, low LOD (down to 25 nM) and fast response time (<2 min) were successfully obtained. (3) More importantly, by integrating **PPAB-Gal**-loaded TLC plate with smart phone-adaptable RGB color (red, green, blue) mode analysis, quantitative and reliable monitoring aquatic product freshness were further realized by direct visualization. (4) **PPAB-Gal** NPs also offered bioimaging application for detection spermine in live cells. 

## 2. Experimental Section

### 2.1. Instruments and Materials

The common organic solvents and other chemicals were commercially available without further purification. 8 BAs including spermine (Spm), spermidine (Spd), putrescine (Put), cadaverine (Cad), tyramine (Tyr), histamine (His), 2-phenylethylamine (Phe) and tryptamine (Try) were obtained from Bide Pharmatech Ltd. (Shanghai, China). All optical spectra were recorded at 25 °C. For NMR, UV-vis and fluorescence measurement, the liquid samples were used. Bruker Avance III 400 MHz (Bruker, Rheinstetten, Germany) was used to provide ^1^H and ^13^C NMR spectra. Hitachi U-3900H spectrophotometer and Hitachi F-4500 spectrophotometer were utilized to supply the absorption and fluorescence spectra, respectively. MALDI-TOF mass and FT-IR spectra were recorded on a Autoflex speed TOF/TOF mass spectrometer (Bruker, Rheinstetten, Germany) and a Brucker VERTEX 80 V FTIR spectrometer (Brucker, Rheinstetten, Germany), respectively. The total volatile base nitrogen (TVBN) content is a key factor in measuring meat quality, which is determined with a Kjeldahl nitrogen detector (BUCHI K-375, Labortechnik, AG, Swiss). 

Compounds **1**, **3** and **PPAB-1** were synthesized according to previous work [35,36]. 

To gain a deep understanding of the noncovalent interactions (π–π stacking, and hydrogen bond) relationship between **PPAB-Gal** and aromatic BAs, the computational platforms such as Gaussian 16, Multiwfn software, VMD program and the independent gradient model based on Hirshfeld partition (IGMH) were utilized.

### 2.2. Synthesis of Compound PPAB-2

**PPAB-1** (100.00 mg, 0.08 mmol) and K_2_CO_3_ solid (29.00 mg, 0.21 mmol) were added to the mixture of CH_2_Cl_2_/MeOH (2/1, *v*/*v*) under nitrogen. After stirring at room temperature for 30 min. The crude product was separated with silica gel column chromatography to yield **PPAB-2** as green solid (61.00 mg, 70%). ^1^H NMR (400 MHz, CDCl_3_) δ 8.98 (d, *J* = 2.2 Hz, 2H), 8.37 (d, *J* = 11.1 Hz, 4H), 7.81 (dd, *J* = 8.7, 2.0 Hz, 2H), 7.31 (d, *J* = 8.8 Hz, 2H), 6.99 (d, *J* = 8.8 Hz, 2H), 4.15 (t, *J* = 6.5 Hz, 4H), 3.26 (s, 2H), 1.90 (p, *J* = 6.7 Hz, 4H), 1.47–1.24 (m, 20H), 0.91 (t, *J* = 6.4 Hz, 6H). HRMS (ESI): *m*/*z* [M+H]^+^ calcd for C_48_H_48_B_2_Br_2_F_4_N_6_O_2_: 998.2457, found: 998.2401.

### 2.3. Synthesis of Compound **PPAB-OAc**

**PPAB-2** (200.00 mg, 0.20 mmol) and compound **3** (300.00 mg, 0.80 mmol), CuSO_4_·H_2_O (100.00 mg, 0.40 mmol) and sodium ascorbate (100.00 mg, 0.50 mmol) were added to the mixture of THF/H_2_O (20.00 mL/8.00 mL). After stirring at room temperature for 7 h, water was added. The reaction mixture was extracted three times with dichloromethane. The organic layer solution was collected, and dichloromethane was removed under reduced pressure. The crude product was separated with silica gel column chromatography to yield **PPAB-OAc** as green solid (348.00 mg, 25%). ^1^H NMR (400 MHz, CDCl_3_) δ 9.01 (d, *J* = 2.2 Hz, 2H), 8.67 (d, *J* = 2.2 Hz, 2H), 8.41 (m, 4H), 8.15 (s, 2H), 7.46 (d, *J* = 8.8 Hz, 2H), 7.03 (d, *J* = 8.9 Hz, 2H), 5.89 (d, *J* = 9.2 Hz, 2H), 5.63–5.52 (m, 4H), 5.29 (dd, *J* = 10.3, 3.3 Hz, 2H), 4.32–4.13 (m, 10H), 2.28 (s, 6H), 2.05 (d, *J* = 10.0 Hz, 12H), 1.93 (s, 6H), 1.55 (q, *J* = 7.3 Hz, 4H), 1.47–1.17 (m, 20H), 0.94–0.88 (m, 6H). HRMS (ESI): *m*/*z* [M+H]^+^ calcd for C_76_H_86_B_2_Br_2_F_4_N_12_O_20_: 1745.4583, found 1745.4657.

### 2.4. Synthesis of Compound **PPAB-Gal**

**PPAB-OAc** (30.00 mg, 0.02 mmol) and K_2_CO_3_ (5.88 mg, 0.04 mmol) was added to dichloromethane/MeOH (9.00 mL/3.00 mL). After stirring for 4 h, green solid was presented. After filtering, the resulting solid was washed with dichloromethane to obtain green solid **PPAB-Gal** (19.40 mg, 80%). ^1^H NMR (400 MHz, DMSO-*d*_6_) δ 9.21 (s, 2H), 8.93 (s, 2H), 8.82 (d, *J* = 2.2 Hz, 2H), 8.66 (dd, *J* = 8.8, 2.0 Hz, 2H), 8.47 (dd, *J* = 8.7, 2.3 Hz, 2H), 7.48 (d, *J* = 8.9 Hz, 2H), 7.36 (d, *J* = 8.9 Hz, 2H), 5.58 (d, *J* = 9.1 Hz, 2H), 5.32 (d, *J* = 5.8 Hz, 2H), 5.11 (d, *J* = 5.5 Hz, 2H), 4.79–4.70 (m, 4H), 4.25 (t, *J* = 6.4 Hz, 4H), 4.13–4.03 (m, 2H), 3.81 (q, *J* = 6.2, 5.6 Hz, 4H), 3.58 (m, 6H), 1.83 (q, *J* = 6.8 Hz, 4H), 1.45–1.22 (m, 20H), 0.92–0.86 (m, 6H). HRMS (ESI): *m/z* [M+H]^+^ calcd for C_60_H_70_B_2_Br_2_F_4_N_12_O_12_: 1408.7136, found: 1408.8127.

### 2.5. Spectroscopic Measurements

**PPAB-Gal** was dissolved in DMSO to give the stock solution (0.1 mM), which was diluted with water to produce test solution (10 μM) in DMSO/H_2_O (4/1, *v*/*v*). Tryptamine stock solution (0.1 M) was prepared in ethanol, and the remaining seven BAs stock solution (0.1 M) were prepared in H_2_O. When **PPAB-Gal** test solution was mixed with BAs solution for desired time, the absorption and fluorescence spectral measurement was carried out in a 2 mL quartz cell with an optical path length of 1 cm at room temperature. 

### 2.6. Fabrication of **PPAB-Gal**-Loaded TLC Plates

**PPAB-Gal** solution (1 mM in DMSO) was prepared. TLC plates were dried at oven at 105 °C for 30 min to remove the possibly remaining water. The dried TLC plates were soaked into **PPAB-Gal** solution for 2 min. Then, TLC plates were taken out and dried in the oven. The same procedure was repeated three times to prepare **PPAB-Gal** TLC plates.

### 2.7. Visual Detection of Shrimp or Tuna Freshness with **PPAB-Gal**-Loaded TLC Plate

Freshly slaughtered samples (shrimp and tuna) were bought from local supermarket (Guangzhou, China). The samples were placed in glass Petri dishes. The **PPAB-Gal**-loaded TLC plates were attached to the inner cover of another Petri dishes. The two Petri dishes were sealed with tape. These Petri dishes with real samples were kept at 25 or 4 °C for different storage time, respectively. Then, the photos were taken under daylight or 365 nm UV lamp by smartphone. The smartphone-adaptable RGB color APP was used to output red, green, blue values. 

### 2.8. Determination of TVBN Content in Shrimp or Tuna

On the base of Standard GB 5009.228-2016 (China) [37], TVBN content in real samples was measured as a control index to evaluate the meat freshness. The shrimp or tuna was purchased from local supermarket. The samples (1 g) were cut into small pieces and 50 mL distilled water was added. After shaking for 30 min and filtration, MgO (1 g) was added. The 25 mL resulting extraction solution was selected as experimental samples. Meanwhile, 50 mL (NH_4_)_2_SO_4_ aqueous solution (0.004 g/mL) was used as a control. TVN value was measure by steam distillation through Kjeldahl distillation unit. The resulting distillate was neutralized by H_3_BO_3_ (30 mL, 20 g/L), followed by titration with HCl (0.1 mol/L). 

### 2.9. Preparation of **PPAB-Gal** NPs

A modified co-precipitation method was used to prepare **PPAB-Gal** NPs. In a typical experiment, 2 mg of **PPAB-Gal** and 5 mg of DSPE-PEG2000 were dissolved in 1 mL THF solution, and the mixture was injected into 5 mL deionized water. Then the mixture was evaporated to remove THF completely on a rotary evaporator at 40 °C. The resulting mixture was cooled to room temperature. The obtained solution of **PPAB-Gal** NPs was filtered by a 0.22 μm filter to discard big aggregates and stored at 4 °C for further use. 

### 2.10. Cell Culture

Cells were cultured in high-glucose Dulbecco’s Modified Eagle’s Medium (H-DMEM) containing 10% fetal bovine serum (FBS) and 1% penicillin streptomycin at 37 °C in a humidified environment containing 5% CO_2_. Before the experiment, the cells were precultured until confluence was reached.

### 2.11. Cell Imaging

HeLa cells were seeded in the 12-well plate and cultured in H-DMEM with 10% FBS at 37 °C in a humidified environment containing 5% CO_2_. After 80% confluence, the medium was removed and the adherent cells were rinsed twice with 1 × PBS. Cells were incubated with **PPAB-Gal** NPs (50 μM) in absence or preence of spermine (100 μM) for 4 h. After washing the culture dishes three times with PBS, fluorescence imaging experiments were carried out on a LSM710 confocal microscope (Carl Zeiss, Germany).

## 3. Results and Discussion

### 3.1. Characterization and Spectra Performance of PPAB-Gal

**Scheme 2:**
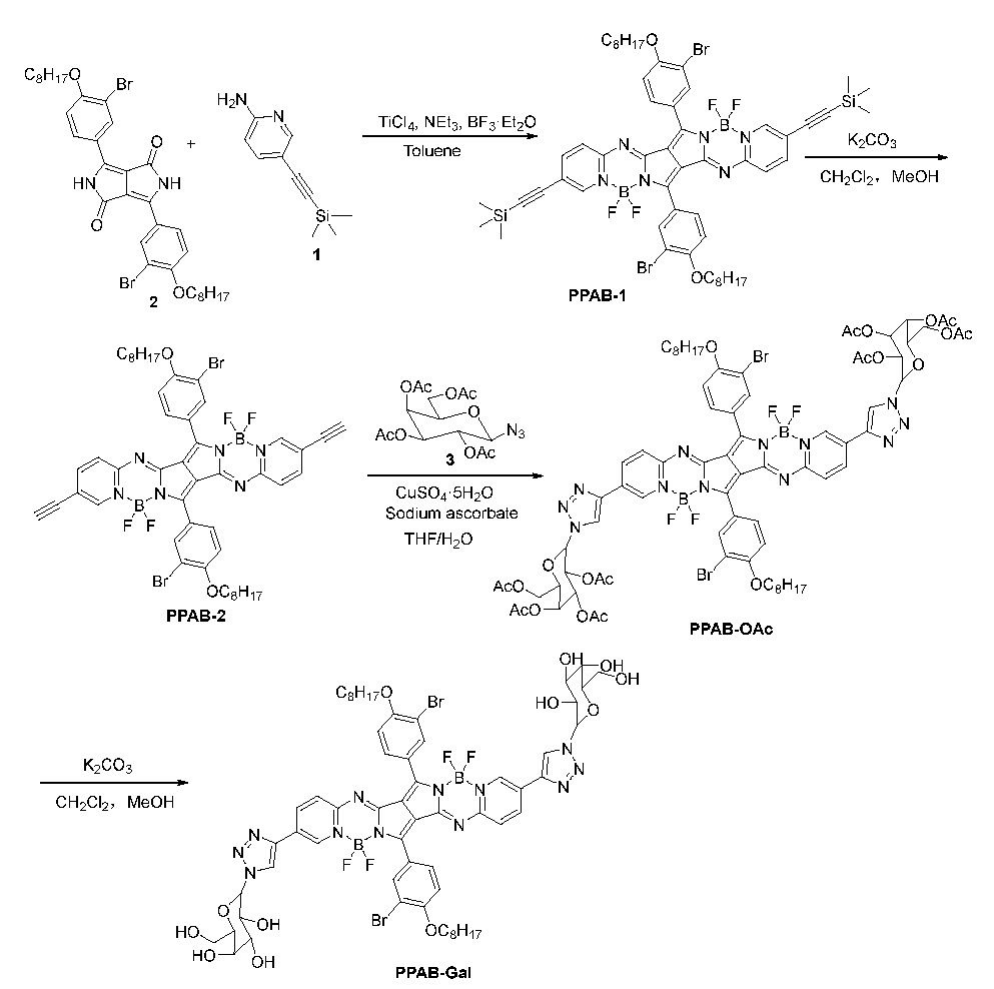
The synthetic routes of **PPAB-Gal**.

In Scheme 2, the condensation reaction between trimethylsilicon-alkynyl aminopyridine (compound **1**) and diketopyrrolopyrrole (compound **2**) and a subsequent boron complexation generated **PPAB-1**. After desilylation reaction of **PPAB-1** with K_2_CO_3_, terminal alkyne containing **PPAB-2** was obtained [35]. The following Cu(I)-catalyzed azide–alkyne click cycloaddition with acetylgalactose-azide (compound **3**) gave **PPAB-OAc**. The deprotection of **PPAB-OAc** in presence of base generated galactose functionalized **PPAB-Gal**. The chemical structures of these compounds were characterized using MALDI-TOF and NMR spectroscopy (Figures S1–S6). The n-octanol/water partition coefficient (Clog P) values were used to evaluate their hydrophilicity. The value of 13.268, 12.928 and 8.641 for **PPAB-2**, **PPAB-OAc** and **PPAB-Gal** was found, respectively, indicating their hydrophilicity was gradually improved by introduction of 1,2,3-triazole and galactose groups to PPAB core. Meanwhile, the presence of galactose made **PPAB-Gal** soluble in polar solvents (acetone, MeOH, EtOH, DMSO, DMF).

As shown in Figure S7a,b, a slightly gradual red-shift from 668, 673 and 675 nm for absorption maximum as well as 686, 692 and 698 nm for emission maximum was shown for **PPAB-2**, **PPAB-OAc** and **PPAB-Gal** in CH_2_Cl_2_, respectively. The results suggested that conjugation of galactose via 1,2,3-triazoles linker did not significantly alter the spectral properties of PPAB dyes. **PPAB-2** and **PPAB-OAc** showed solvent polarity independent absorption and emission maximum (Figure S8a–d and Table S1). Interestingly, **PPAB-Gal** exhibited a slightly solvent-dependent absorption and emission behavior due to the presence of polar galactose groups (Figure S8e,f). The changes of absorption maximum from 660 to 676 nm as well as emission maximum from 679 to 698 nm from MeOH to DMSO were observed. It is possible the galactose of **PPAB-Gal** was susceptible to solvent polarity, making the spectral properties different. The density functional theory (DFT) calculations showed that the energy gaps (*E_g_*) of 2.027, 2.010 and 2.032 eV for **PPAB-2**, **PPAB-OAc** and **PPAB-Gal**, respectively (Figure S9). The electron densities were mainly distributed on the PPAB core for three PPAB dyes, which was consistent with their minor solvatochromic behavior.

The aggregation behavior of **PPAB-2**, **PPAB-OAc** and **PPAB-Gal** were investigated. In DMSO, **PPAB-2** showed a strong shoulder absorption peak at 615/669 nm. Upon adding 15% water volumetric factions (*f*_w_), the absorption intensity significantly decayed, and the spectra became broader. The red-shifted shoulder at 627/679 nm was observed when *f*_w_ increased to 90% (Figure S7c), indicative of formation of *J*-aggregates. For **PPAB-OAc**, similar absorption decay and broadening was shown when *f*_w_ increased from 0 to 90% (Figure S7e). These observations indicated that **PPAB-2** and **PPAB-OAc** with planar rigid π-conjugated structures exhibited a “head-to-tail” orientation and parallel sliding structure to form *J*-aggregates. For **PPAB-Gal**, the presence of 15% *f*_w_ didn’t induce the change of absorption spectrum. If *f*_w_ increased to 90%, the original absorption shoulder at 619/676 nm was blue-shifted to 596 /671 nm, indicating the *H*-aggregates were formed (Figure S7g). This phenomenon can be ascribed to the presence of hydrophilic galactose of **PPAB-Gal**. It’s possible that the multiple H-bonds between galactoses potentially increase “face-to-face” stacks among **PPAB-Gal** aggregated state, thereby facilitating *H*-aggregates formation. Some studies revealed typical non-covalent bonds contributed to the self-assembly process [38]. 

In the emission spectra, **PPAB-2** showed more obvious aggregation-caused quenching (ACQ) effect than **PPAB-OAc** in presence of water. Upon addition of 15% *f*_w_, the fluorescence quenching rate of 99% and 70% was found for **PPAB-2** and **PPAB-OAc**, respectively (Figure S7d,f). On the contrary, **PPAB-Gal** showed a certain anti-aggregation effect. There were no fluorescence quenching phenomena when *f*_w_ was lower than 15%. However, the emission intensity at 698 nm was largely weakened as *f*_w_ increased from 15 to 30% (Figure S7h). Then the fluorescence was completely disappeared by adding more water. This phenomenon was agreement with their Clog P values, where more hydrophobic **PPAB-2** and **PPAB-OAc** resulted in a higher degree of aggregation and drastic fluorescence quenching in an aqueous solution. All these results indicated the presence of hydrophilic galactose played a significant role in adjusting photophysical properties and the formation of *H*-aggregates of **PPAB-Gal**.

### 3.2. Spectral Response of PPAB-Gal Towards 8 BAs

**Figure 1:**
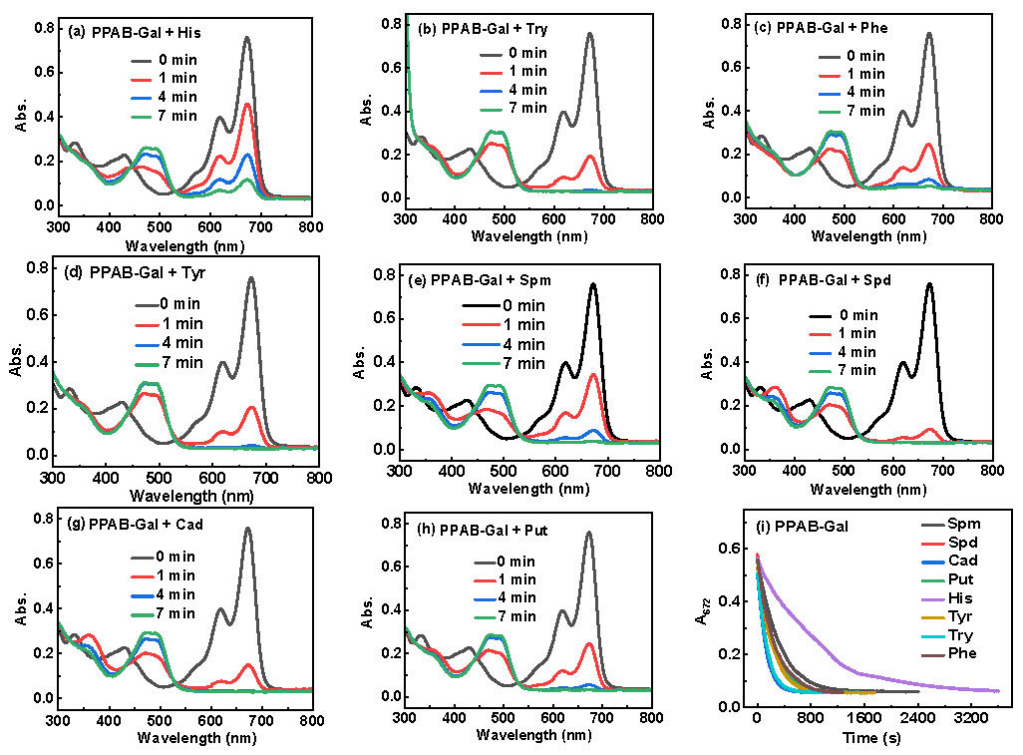

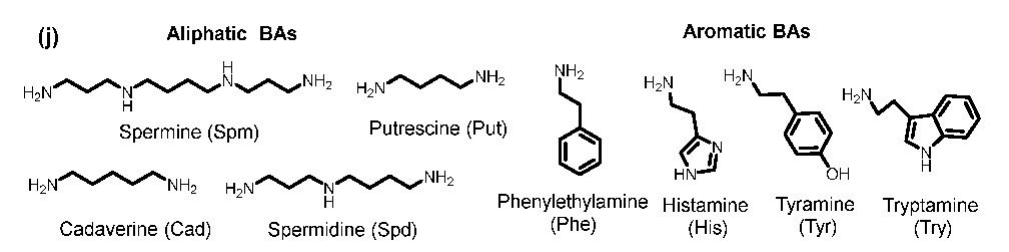
Time-dependent of UV-vis spectra of **PPAB-Gal** (10 μM) in presence of (**a**) His, (**b**) Try, (**c**) Phe, (**d**) Tyr, (**e**) Spm, (**f**) Spd, (**g**) Cad, and (**h**) Put (200 μM) in DMSO/H_2_O (4/1, *v*/*v*). (**i**) The kinetic curves of **PPAB-Gal** in presence of 8 BAs. (**j**) The chemical structures of eight BAs.

Among aromatic BAs, high intake of toxic His could cause poisoning and allergic reactions. His was used as a typical aromatic BA model to test the sensing practicability of **PPAB-Gal** in DMSO/H_2_O (4/1, *v*/*v*). As indicated in Figure 1a, time-dependent absorption spectra of **PPAB-Gal** in presence of His was measured. Delightedly, the characteristic shoulder peak at 619/673 nm in NIR region showed a rapid drop along with the increment of reaction time. Moreover, a new absorption shoulder band at 470/483 nm gradually emerged, indicating less conjugated new compounds were formed. The aromatic BAs (His, Try, Tyr, Phe) and aliphatic BAs (Spd, Spm, Put, Cad) induced similar UV-vis spectra changes (Figure 1b–h,j). The reaction kinetics curves indicated that **PPAB-Gal** possessed high reaction rate towards eight BAs and quickly reached equilibrium within 10 min (Figure 1i). The pseudo-first-order rate constant (*k*_obs_) was calculated to be 3.151, 8.221, 8.097, 4.371, 1.127, 4.615, 7.057, and 3.893 × 10^−3^ s^−1^ for Spm, Spd, Cad, Put, His, Tyr, Try, and Phe, respectively (Figure S10). Unexpectedly, Try had higher reaction rate than Spm and Put. Among them, His showed the most sluggish reaction rate towards **PPAB-Gal**.

**Figure 2:**
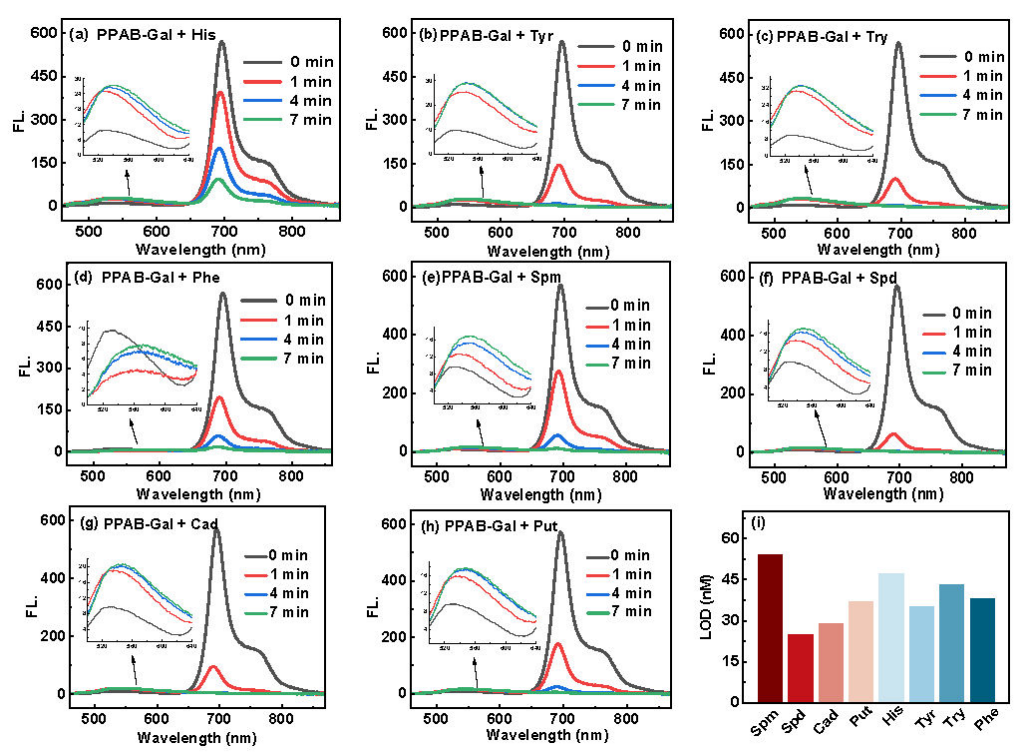
Time-dependent of emission spectra of **PPAB-Gal** (10 μM) in presence of (**a**) His, (**b**) Tyr, (**c**) Try, (**d**) Phe, (**e**) Spm, (**f**) Spd, (**g**) Cad, and (**h**) Put (200 μM) in DMSO/H_2_O (4/1, *v*/*v*). (**i**) The LOD values of detection of 8 BAs using fluorescence method.

Time-dependent fluorescence spectra of **PPAB-Gal** after adding aromatic BAs (His, Try, Tyr, Phe) and aliphatic BAs (Spd, Spm, Put, Cad) were shown in Figure 2a–h. Obviously, with the increment of reaction time, fluorescence intensity at 695 nm reduced dramatically, and those of a new emission peak at 545 nm enhanced gradually. As a result, I_545_/I_695_ values enhanced with the reaction time from 0 to 10 min and then kept invariable. In the case of Tyr and Try, signal changes reached maximum after 4 min. In summary, **PPAB-Gal** showed similar reactivity towards aliphatic BAs and aromatic BAs, mainly owing to additional noncovalent interactions (π–π stacking, and hydrogen bond, etc.).

**Figure 3:**
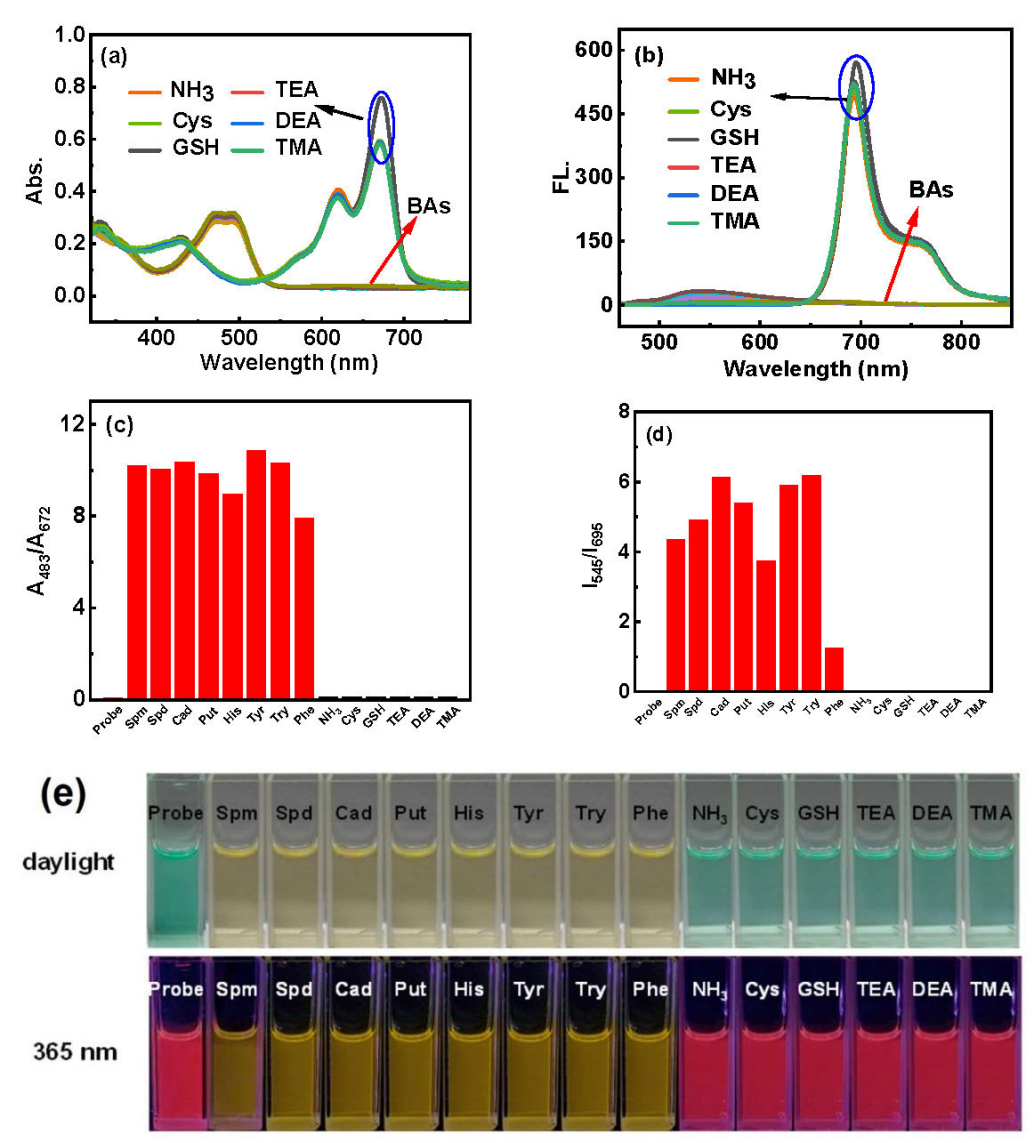
The (**a**) UV-vis absorption, (**b**) emission spectra, corresponding (**c**) A_483_/A_672_ and (**d**) A_545_/A_695_ value of **PPAB-Gal** (10 μM) in presence of 8 BAs and other analytes (200 μM) in DMSO/H_2_O (4/1, *v*/*v*). (**e**) The photos of **PPAB-Gal** (10 μM) in presence of BAs and other analytes (200 μM) under daylight and 365 nm irradiation.

For the selectivity experiments, Cys, GSH, triethylamine, diethylamine, trimethylamine, and NH_3_ were selected. Other amines and biothiols didn’t show obvious absorption and fluorescence spectral changes of **PPAB-Gal** except eight BAs (Figure 3a,b). As a result, only eight BAs induced distinct A_483_/A_672_ and I_545_/I_695_ enhancement (Figure 3c,d). The selective ratiometric color change from green to faint yellow and fluorescence changes from red to orange were observed, which allowed visual detection of eight BAs (Figure 3e). 

The absorption and emission spectra of **PPAB-Gal** in presence of BAs showed concentration-dependent (Figures S11 and S12). The value of A_672_ exhibited a good linear relationship with the BAs content at 0–180 μM. The limit of detection (LOD) of His (250 nM), Tyr (120 nM), Try (110 nM), Phe (180 nM), Spm (210 nM), Spd (120 nM), Cad (110 nM), Put (140 nM) was shown based on 3δ/k method. In addition, the good linear relationship between I_695_ and BAs concentration at 0–140 μM was presented with the lower LOD value of His (47 nM), Spm (54 nM), Spd (25 nM), Cad (29 nM), Put (37 nM), Tyr (35 nM), Try (43 nM), Phe (38 nM), as summarized in Figure 2i. Comparing with reported fluorescence systems for BAs sensing (Table S2), **PPAB-Gal** presented several significant advantages: (1) fast and efficient sensing aromatic BAs; (2) large blue shifts (Δλ_ab_ = 130 nm, Δλ_em_ = 150 nm) with colorimetric and ratiometric fluorescence signals (3) simultaneous response toward 8 BAs (4 aromatic BAs and 4 aliphatic BAs) for higher sensitivity. For instance, there were some reports on fluorescent detection for His with molecularly imprinted polymer or ZnO quantum dots (LOD: 1 μM or 0.97 μM, respectively) [39,40], as well as colorimetric detection of His with gold nanoparticles (LOD: 0.43 μM with an incubation time of 4 h) [41,42], Therefore, **PPAB-Gal** could recognize the presence of BAs in food samples since it exhibited unique sensitivity to typical 8 BAs.

### 3.3. The Effect of Galactose Group on Sensing Performance of **PPAB-Gal**

To get insight into the role of noncovalent interactions (hydrogen bond and π–π interaction) of **PPAB-Gal** for BAs sensing, **PPAB-2** with solely PPAB core and **PPAB-OAc** with 1,2,3-triazole ring and PPAB core were used as control probes. As shown in Figure S13 and S14, we can similarly observe decay of NIR absorption band in presence of eight BAs, signifying the importance of the existence of the PPAB core. However, they showed much slower reaction rate than **PPAB-Gal**. For example, the decay value at 673 nm of 99%, 19%, 12% was found for **PPAB-Gal**, **PPAB-OAc** and **PPAB-2**, respectively. Meanwhile, the new absorption band at 470/483 nm was also much weaker, suggesting less new compounds were formed in **PPAB-2** and **PPAB-OAc** than those in **PPAB-Gal**. It was obvious that **PPAB-Gal** showed the highest reactivity towards eight BAs than **PPAB-2** and **PPAB-OAc**. The corresponding data are summarized in Figure S15a. The reactivity towards BAs was shown in this order: **PPAB-Gal** >> **PPAB-OAc** > **PPAB-2**. 

To understand the response priority of **PPAB-Gal** and other probes (**PPAB-2**, **PPAB-OAc**) to BAs, electrostatic surface potential (ESP) maps were carried out, which can provide additional information of evaluating chemical reactivity. In Figure S15b, the electrostatic potential area in the blue area of N, F, and O atoms of three molecules was negative, which was susceptible to nucleophilic attack. Moreover, **PPAB-Gal** possessed the highest electronegativity compared to **PPAB-2** and **PPAB-OAc**, leading to the fastest reaction rate towards BAs. All results demonstrated the importance of the existence of the 1,2,3-triazole moiety and galactose for enhanced reactivity, providing the feasibility of **PPAB-Gal** for rapid evaluation of 8 BAs. 

### 3.4. Sensing Mechanism

**Figure 4:**
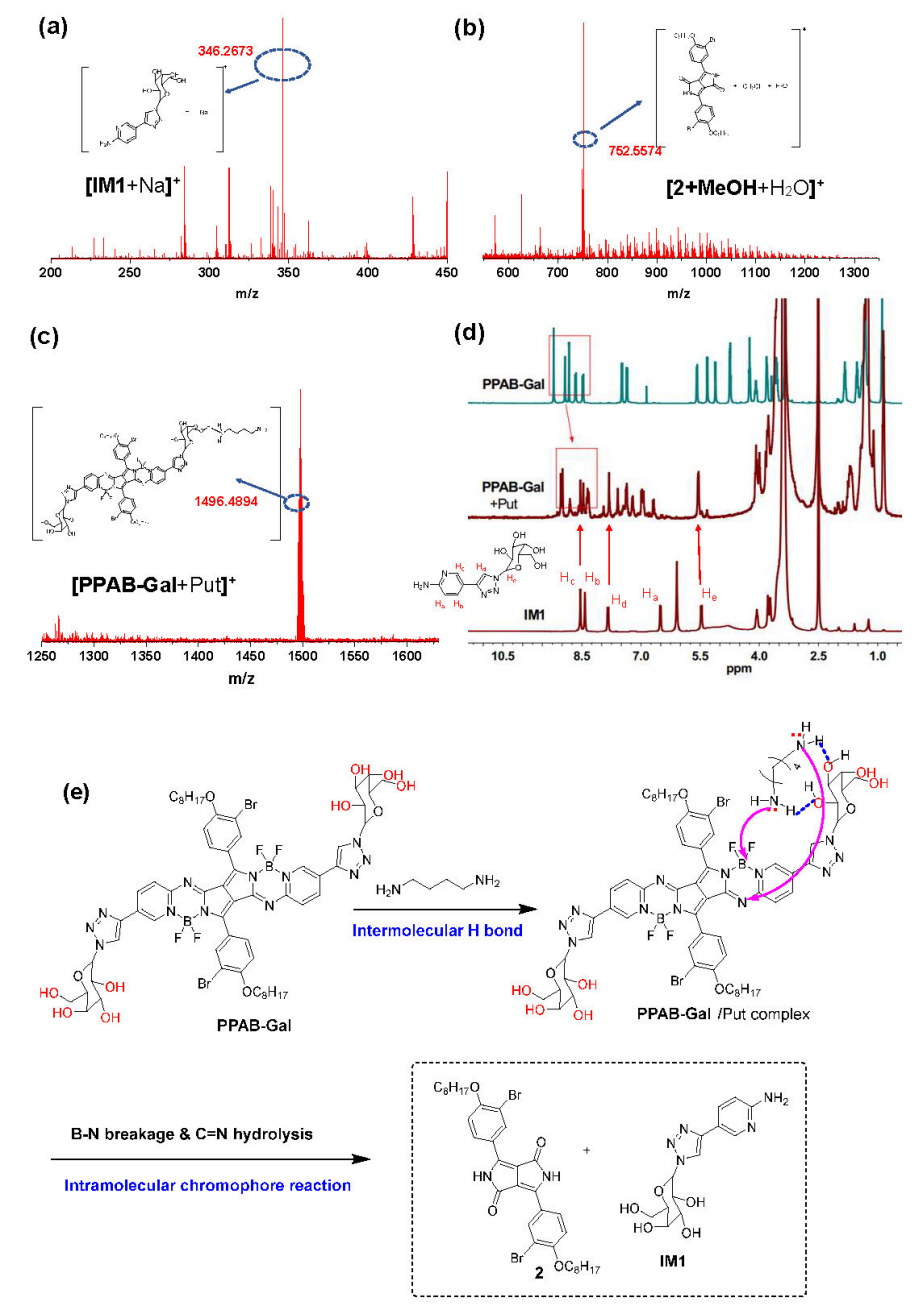
(**a**–**c**) The HRMS spectra of **PPAB-Gal** in presence of less Put. (**d**) The ^1^H NMR spectra of **PPAB-Gal** in absence and presence of Put, and control compound **IM1**. (**e**) The possible sensing mechanism between **PPAB-Gal** and Put.

The specific response mechanism of **PPAB-Gal** to BAs (Put as a BA model) was confirmed by HRMS, ^1^H NMR and FT-IR spectra. As shown in Figure 4a–c, a prominent peak at *m/z* = 1496.4894 corresponding to the **PPAB-Gal**/Put complex was found, indicative of the presence of noncovalent interactions between **PPAB-Gal** and Put. The reaction products of galactose group functionalized alkynyl aminopyridine **IM1** (346.2673 for [**IM1**+Na]^+^) and the diketopyrrolopyrrole compound **2** (752.5574 for [**2**+MeOH+H_2_O]^+^) were also detected. In addition, ^1^H NMR spectrum showed that after the addition of Put, the chemical shift of aromatic hydrogen region of **PPAB-Gal** shifted to high field (Figure 4d). The possible reason was ascribed that the strong electron withdrawing PPAB core was destroyed, and less conjugated compounds with enhanced electron cloud density were shown. Moreover, the characteristic peaks of **IM1** can be found in **PPAB-Gal**+Put system. Meanwhile, the FT-IR spectra showed that the new stretching peaks at 1714, 1630, 3853 and 3742 cm^−1^ were assigned the vibrational frequencies of C=O and N-H of compounds **2** and **IM1** (Figure S16). The energy gap of **PPAB-Gal** was calculated to be 2.03 eV, while the energy gaps of the reaction products (compounds **2** and **IM1**) were increased to 2.64 eV and 5.22 eV, respectively, which coincided with the blueshift of the absorption and emission spectra of **PPAB-Gal** in response to Put (Figure S17). 

To further reveal the sensing mechanism of **PPAB-Gal** and aromatic BAs, the bond critical point (BCP) of the hydron-bond of interest was analysed [43]. Taking Phe as an aromatic BAs example, the HO…HN hydrogen bond interaction (1.82 Å) represented strong interactions between the galactose moiety of **PPAB-Gal** and amine group of Phe. Moreover, the weak multiple hydrogen bond interactions with C-H…N (3.33–3.58Å) from phenyl of Phe and N atom of 1,2,3-triazole and strong π…π interaction between phenyl of Phe and 1,2,3-triazole ring of **PPAB-Gal** were observed (Figure S18a). The BCP value of 0.04381 and 0.00482 for HB and π…π interaction was found. Similarly, the hydron-bond and π…π interaction were also found between **PPAB-Gal** and His (or Try), as shown in Figure S18b,c. All in all, these noncovalent interactions facilitated to bring aromatic BAs to the proximity of the **PPAB-Gal**, which was beneficial for chemical reaction based fluorescent probes with improved sensing efficiency. In the same way, the aliphatic BAs would approach **PPAB-Gal** through H-bonds. Therefore, it is reasonable that **PPAB-Gal** showed similar reactivity towards aromatic and aliphatic BAs. 

Based on discussion mentioned above, the possible reaction mechanism between **PPAB-Gal** and Put was shown in Figure 4e, confirming the noncovalent interactions facilitated chromophore reaction. Namely, the noncovalent interactions between **PPAB-Gal** and Put would generate **PPAB-Gal**/Put complex, following intramolecular chromophore reaction to produce a less conjugated diketopyrrolopyrrole (compound **2**) and galactose group functionalized alkynyl aminopyridine (**IM1**). 

### 3.5. **PPAB-Gal**-Loaded TLC Plates Toward Put and Cad Vapor

**Figure 5:**
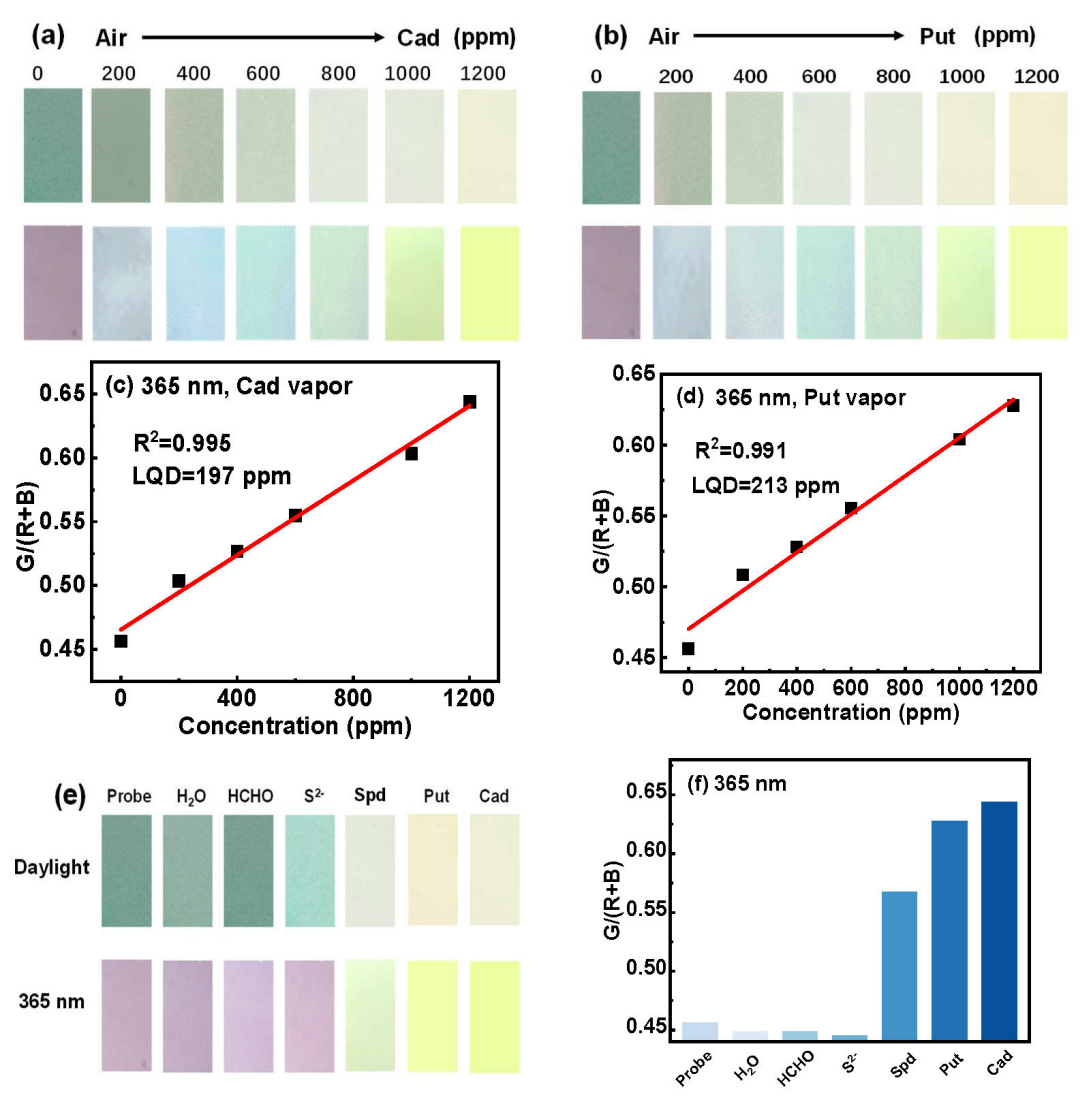
The photographs of **PPAB-Gal**-loaded TLC plate in presence of (**a**) gaseous putrescine, (**b**) cadaverine and (**e**) other analytes under daylight and 365 nm irradiation. (**c**,**d**,**f**) The calibration curve of G/(R+B) value extracted from the photos (**a**,**b**,**e**) taken with Color Finder APP, respectively.

The release of volatile Cad and Put produces bad-tasting food and foul-smelling odor during food spoilage process. The excessive intake can result in hypotension, allergies, headaches, even shock or death [44]. However, volatile amines are generally colorless, which makes their direct visual detection of various amines in environmental samples highly difficult. In order to develop a portable sensing platform for on-site measurement of Put and Cad vapor, TLC plate was immersed in the **PPAB-Gal** solution and dried in air. After **PPAB-Gal**-loaded TLC plates were exposed to Cad vapor for 10 min, the images were recorded with a smartphone in different conditions. As shown in Figure 5a, the remarkable color and fluorescence response after exposure to various concentrations of Cad vapors was displayed. A color recognizer APP with RGB (red, green, blue) was utilized to analyse the fluorescence images. The excellent linear relationship (R^2^ = 0.995) between value of G/(R+B) was and the Cad vapor concentration at 0–1200 ppm was found (Figure 5c). The limit of quantitation detection (LQD) value of 197 ppm was found. Similarly, **PPAB-Gal**-loaded TLC plates displayed distinct color and emission changes upon exposure of Put vapor, leading to LQD value of 213 ppm (Figure 5b,d). 

The selectivity of the **PPAB-Gal**-loaded TLC plates to BAs vapor against other gaseous analytes was also investigated. In Figure 5e, obvious color and emission changes after exposure to Spd/Cad/Put vapors were shown, whereas no changes were displayed in the presence of other vapor (H_2_O, HCHO, H_2_S). The higher value of G/(R+B) under 365 nm irradiation for Spd/Cad/Put vapors against other analytes indicated **PPAB-Gal** TLC plates could be used as an ideal and portable sensing platform for selective and visual sensing of BAs vapor (Figure 5f). 

### 3.6. Visually Monitoring the Freshness of Tuna and Shrimp

**Figure 6:**
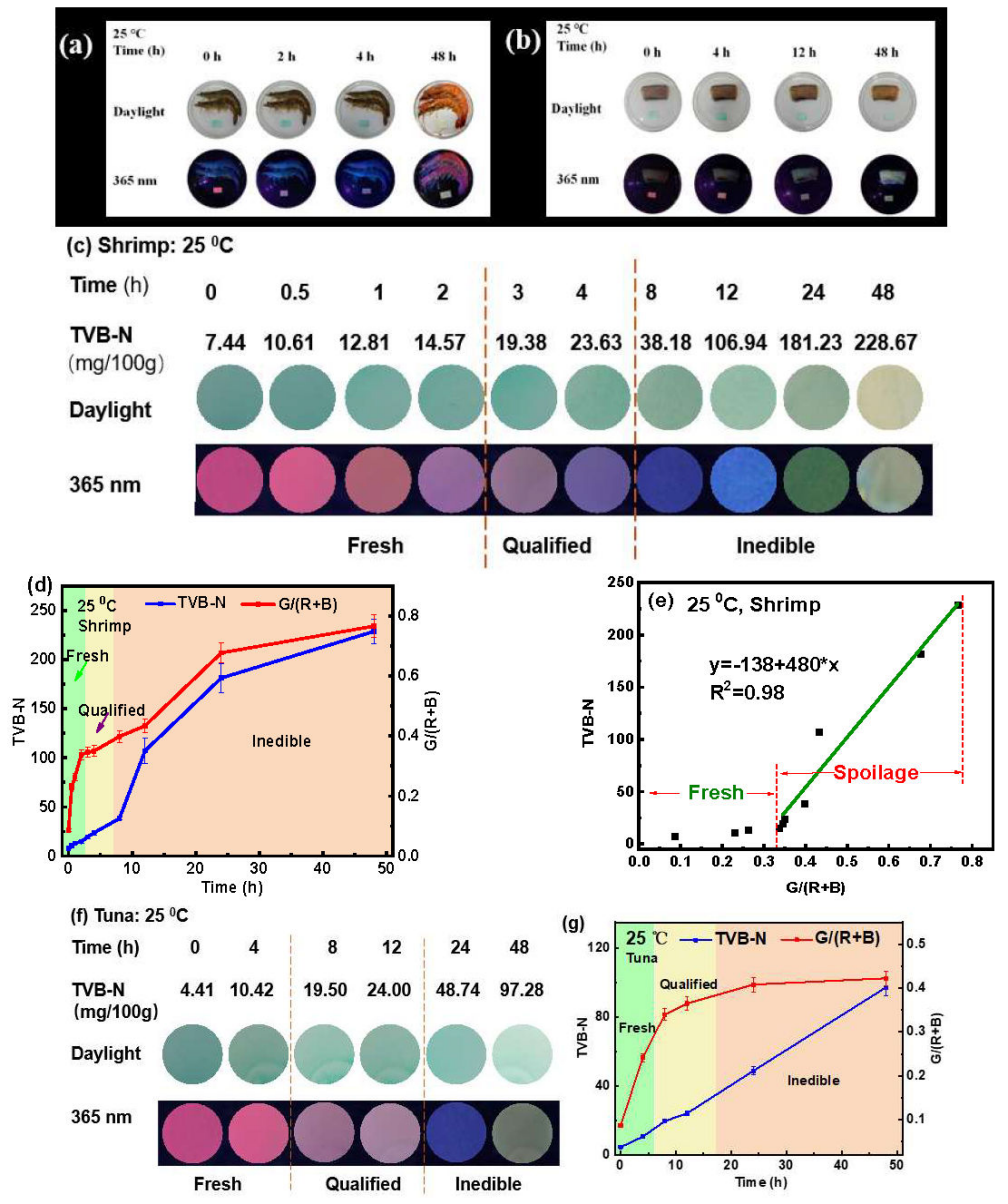
(**a**) The design strategy of visual, and non-destructive sensing platform based on **PPAB-Gal**-loaded TLC plate. (**b**) The color change and emission of **PPAB-Gal**-loaded TLC plate for shrimp (**a**) and tuna (**b**) at 25 °C, respectively. (**c**) The relationship between TVBN value and color change for **PPAB-Gal**-loaded TLC plate for shrimp at 25 °C. The grades of (**d**,**e**) shrimp and (**f**,**g**) tuna freshness evaluation by measuring TVB-N value using a smartphone sensing platform based on the color of **PPAB-Gal**-loaded TLC plate under UV light and shrimp samples stored at 25 °C.

Due to coexistence of multiple components with similar structure and the complex detection surrounding in food, it is demanding to detect BAs in spoiled food owing to their low concentration and mixtures of multiple amines. Based on superior sensing performance of **PPAB-Gal** towards 8 BAs, **PPAB-Gal**-loaded TLC plate was used for in situ monitoring of the freshness of aquatic product (Figure 6a). The visually changes of color and fluorescence photos of the **PPAB-Gal**-loaded TLC plate with shrimp or tuna at 25 °C were exhibited (Figure 6b). With the prolonging storage time, the emission change from red to yellow and color change from green to light yellow were clearly distinguished. On the contrary, no such obvious changes were found for shrimp or tuna at 4 °C (Figures S19 and S20). 

Total volatile basic nitrogen (TVBN) value as the comparative method was utilized to predicate the relationship between **PPAB-Gal**-loaded TLC plate based fluorescence color and sample freshness [45,46,47]. The guidance level of TVBN has been set with ≥20 mg/100 g, 15–20 mg/100 g, ≤15 mg/100 g for spoilage, medium fresh, and freshness, respectively [48]. Figure 6c–e showed the change of TVBN and G/(R+B) value in shrimp after storage for different time at 25 °C. The initial TVB-N value of 7.44 mg/100 g increased to 10.61, 12.81 and 14.57 mg/100 g after 0.5, 1, 2 h storage time, which was identified as fresh. In the meantime, **PPAB-Gal** TLC plate displayed obvious emission color change from red to purple. It cost 8 h to get spoilage with increased TVBN value of 38.18 mg/100 g. There was strong correlation between TVBN and G/(R+B). The good linear relationship between the G/(R+B) value of **PPAB-Gal** TLC plate photos and TVBN value in shrimp (R^2^ = 0.98) was shown. As a result, it’s convenient to evaluate the shrimp spoilage quantitatively with connection between a standard quantitative index of TVBN value and fluorescence signals. 

In the case of tuna, similar fluorescence changes and relationship between TVBN and G/(R+B) were found (Figure 6f,g). However, it cost 24 h to get spoilage with increased TVB-N value of 48.74 mg/100 g at 25 °C. These results verified that **PPAB-Gal**-loaded TLC plate could be utilized as a portable and delicate sensing platform for on-site, non-destructive, convenient and quantitative assessing of aquatic product freshness. 

### 3.7. Visually Monitoring Spermine in Living Cells 

**Figure 7:**
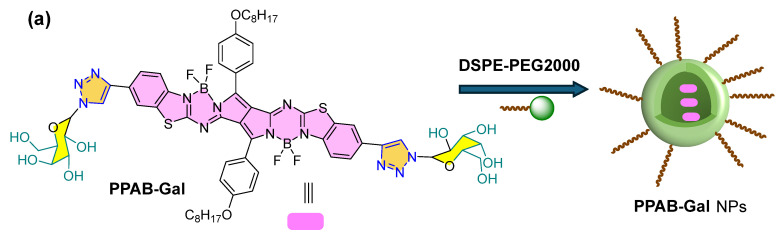

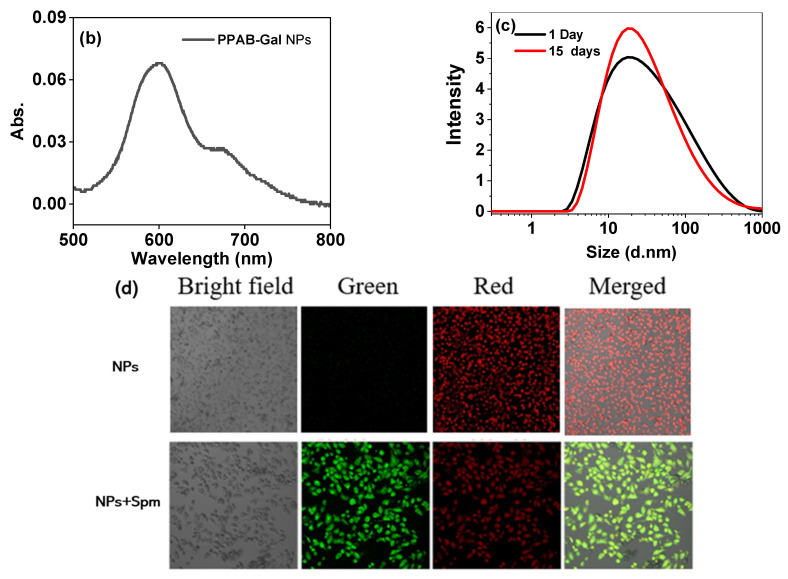
(**a**) Preparation of **PPAB-Gal** NPs. (**b**) UV-vis and (**c**) DLS spectra of **PPAB-Gal** NPs. (**d**) CLSM images of HeLa cells incubated with **PPAB-Gal** NPs (50 μM) in absence and presence of spermine (100 μM) (scale bar: 20 μm).

**PPAB-Gal** nanoparticles was fabricated with DSPE-PEG2000 as a wrapping agent to improve its water solubility (Figure 7a). **PAB-Gal** NPs had NIR absorption band at 600/673 nm and hydrodynamic size of 47 nm through dynamic light scattering (DLS) measurement (Figure 7b,c). The size did not exhibit any changes over 15 days in water, indicative of excellent size stability. Due to presence of galactose group, the excellent biocompability was shown, where the survival rates of Hela cells are larger than 90%.

As mentioned above, **PPAB-Gal** showed high sensitivity to spermine. Since spermine is over-expressed in some cancer cells for assisting cell growth and proliferation, we preliminary investigated whether **PPAB-Gal** NPs could monitor the presence of endogenous spermine in living cells. As shown in Figure 7d, **PPAB-Gal** NPs showed red emission by CLSM imaging. Upon addition of spermine, the green emission was clearly presented, yielding the ratiometric emission signal. This can be ascribed to the chemical reaction between **PPAB-Gal** NPs and spermine to generate the less-conjugated products. These results further demonstrate that **PPAB-Gal** NPs can detect spermine in living cells by ratiometric CLSM signal.

## 4. Conclusions

In summary, we successfully developed **PPAB-Gal** capable of highly efficient detection of eight BAs with significant colorimetric and fluorescence responses by noncovalent interactions enhanced chromophore reaction. The distinctly dual signal changes (Δλ_ab_ = 130 nm, Δλ_em_ = 150 nm), low LOD (down to 25 nM) and fast response time (<2 min) were obtained. The excellent sensing performance enabled **PPAB-Gal**-loaded TLC plate to act as an efficient platform for gaseous Put and Cad sensing and quantitative assessing of the aquatic product freshness through smartphone-adaptable chromaticity diagram. Moreover, **PPAB-Gal** NPs could detect spermine in living cells by ratiometric CLSM signal This work provides a portable and delicate on-site platform for quantitatively estimating of food freshness based on a novel strategy for highly sensing eight BAs. 

## Data Availability

Data contained within the article or Supplementary Materials.

## Supplementary Materials

The following supporting information can be downloaded at: https://www.mdpi.com/article/10.3390/bios15080542/s1, Figure S1: 1H NMR spectrum of PPAB-2 in CDCl3; Figure S2: The TOF spectrum of PPAB-2; Figure S3: 1H NMR spectrum of PPAB-OAc in CDCl3; Figure S4: The TOF spectrum of PPAB-OAc; Figure S5: 1H NMR spectrum of PPAB-Gal in DMSO-d6; Figure S6: The TOF spectrum of PPAB-Gal; Figure S7: (a,b) Normalized absorption and emission spectra of PPAB-2, PPAB-OAc, PPAB-Gal in CH_2_Cl_2_. Absorption and emission spectra of PPAB-2 (c,d), PPAB-OAc (e,f), PPAB-Gal (g,h) in DMSO/H_2_O mixture with different fraction of H_2_O (f,w); Figure S8: Normalized absorption and emission spectra of PPAB-2 (a,b), PPAB-OAc (c,d), and PPAB-Gal (e,f) in different solvents; Figure S9: The DFT results of PPAB-2, PPAB-OAc and PPAB-Gal; Figure S10: The reaction kinetic PPAB-Gal (10 μM) in presence of (a) Spm, (b) Spd, (c) Cad, (d) Put (e) His, (f) Try, (g) Tyr, (h) Phe (200 μM) in DMSO/H_2_O (4/1, *v*/*v*); Figure S11: (a–p) The concentration-dependent UV-vis spectra and linear plot of PPAB-Gal in presence of 8 BAs in DMSO/H_2_O (4/1, *v*/*v*). (q) The LOD values of 8 Bas; Figure S12: (a–p) The concentration-dependent emission spectra and linear plot of PPAB-Gal in presence of 8 BAs in DMSO/H_2_O (4/1, *v*/*v*); Figure S13: Time-dependent of absorption spectra of PPAB-2 (10 μM) in presence of (a) Spm, (b) Spd, (c) Cad, (d) Put, (e) Try, (f) Tyr, (g) Phe and (h) His (200 μM) in DMSO/H_2_O (4/1, *v*/*v*); Figure S14: Time-dependent of UV-vis absorption spectra of PPAB-OAc (10 μM) in presence of (a) Spm, (b) Spd, (c) Cad, (d) Put (e) Try, (f) Tyr, (g) Phe, and (h) His (200 μM) in DMSO/H_2_O (4/1, *v*/*v*); Figure S15: (a) The reaction kinetics curves of PPAB-Gal towards 8 BAs. (b) The electrostatic surface potential (ESP) maps of PPAB-2, PPAB-OAc, and PPAB-Gal; Figure S16: The FT-IR spectra of PPAB-Gal in presence of Put; Figure S17: The DFT results of PPAB-Gal and possible products (compounds 2 and IM1); Figure S18: Optimized molecular structure (gaseous state) of PPAB-Gal and Phe, Try and His with selected interatomic distances (Å); Figure S19: (a,b) The color change and emission of PPAB-Gal -loaded TLC plate for shrimp at 4 °C. (c) The grades of shrimp freshness evaluation by measuring TVB-N value using a smartphone sensing platform based on the color of PPAB-Gal -loaded TLC plate under UV light at 4 °C; Figure S20: (a,b) The photos of color change and emission of PPAB-Gal -loaded TLC plate for tuna at 4 °C. (c) The grades of tuna freshness evaluation by measuring TVB-N value using a smartphone sensing platform based on the color of PPAB-Gal-loaded TLC plate under UV light at 4 °C. Table S1:The photophysical data of PPAB-2, PPAB-OAc and PPAB-Gal in different solvents; Table S2: The comparison of fluorescent probes for BAs detection in food. Table S3: Summary of different BAs detection methods.
