# Supplementary material for: A Galactose-Functionalized Pyrrolopyrrole Aza-BODIPY for Highly Efficient Detection of Eight Aliphatic and Aromatic Biogenic Amines: Monitoring Food Freshness and Bioimaging
Source: Biosensors (Basel). 2025 Aug 18;15(8):542. doi: 10.3390/bios15080542 (PMC12384495; doi:10.3390/bios15080542)
Supplement: Supplementary file 1 [file biosensors-15-00542-s001.zip › biosensors-3761459-supplementary.pdf]

biosensors

Supplementary Materials

A Galactose-Functionalized Pyrrolopyrrole *Aza*-BODIPY for Highly Efficient Detection of Eight Aliphatic and Aromatic Biogenic Amines: Monitoring Food Freshness and Bioimaging

Yujing Gan [1], Bingli Lu [1], Jintian Zhong [1], Xueguagn Ran [2], Derong Cao [1] and Lingyun Wang *[1]

[1] School of Chemistry and Chemical Engineering, South China University of Technology, 381 Wushan Road, Guangzhou, China; 202220122873@mail.scut.edu.cn (Y.G.); binglilu2020@163.com (B.L.); bingzeclassic@outlook.com (J.Z.); drcao@scut.edu.cn (D.C.)
[2] The State Key Laboratory of Swine and Poultry Breeding Industry, Key Laboratory of Animal Nutrition and Feed Science in South China, Ministry of Agriculture and Rural Affairs, Guangdong Provincial Key Laboratory of Animal Breeding and Nutrition, Institute of Animal Science, Guangdong Academy of Agricultural Sciences, Guangzhou, China; rxg59@aliyun.com (X.R.)
* Correspondence: lingyun@scut.edu.cn

1. Experimental Section

1.1. Synthesis of Compound IM1

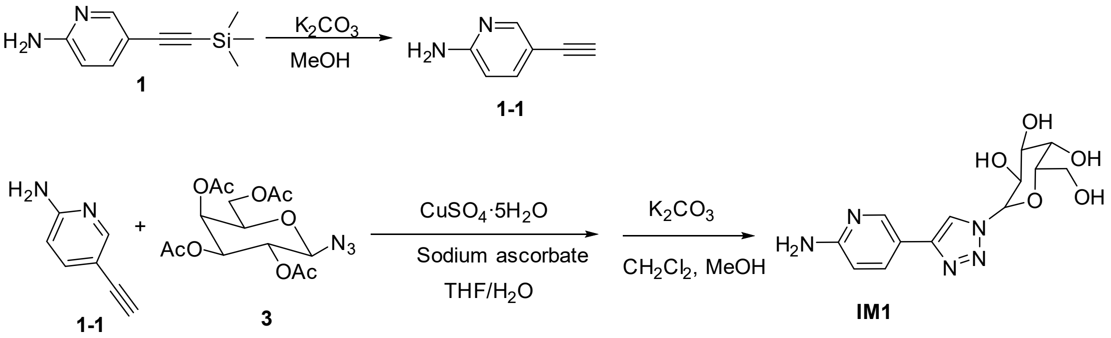

Figure S1 The synthesis of **IM1**.

Received: 1 July 2025
Revised: 25 July 2025
Accepted: 31 July 2025
Published: date

Citation: Gan, Y.; Lu, B.; Zhong, J.; Ran, X.; Cao, D.; Wang, L. A galactose-functionalized pyrrolopyrrole *aza*-BODIPY for highly efficient detection of eight aliphatic and aromatic biogenic amines: monitoring food freshness and bioimaging. *Biosensors* **2025**, *12*, x. https://doi.org/10.3390/xxxxx

Copyright: © 2025 by the authors. Submitted for possible open access publication under the terms and conditions of the Creative Commons Attribution (CC BY) license (https://creativecommons.org/licenses/by/4.0/).

Compound **1** (3.50 g, 18.81 mmol) and potassium carbonate (2.65 g, 19.19 mmol) were added to methanol and stirred for 3 h. After the reaction was completed, the mixture was concentrated under reduced pressure. The crude product was purified by column chromatography to yield compound **1-1** as white solid (2.00 g, 90 %). ^{1}H NMR (400 MHz, Chloroform-d) δ 8.22 (d, J = 2.2 Hz, 1H), 7.50 (dd, J = 8.5, 2.3 Hz, 1H), 6.43 (d, J = 8.5 Hz, 1H), 4.64 (s, 2H), 3.05 (s, 1H).

Compound **1-1** (100 mg, 0.84 mmol), compound **3** (0.63 g, 1.69 mmol), $CuSO_4·H_2O$ (0.42 g, 1.69 mmol) and sodium ascorbate (0.42 g, 2.11 mmol) were added to the mixture of THF (20.00 mL) and water (8.00 mL). After stirring at room temperature for 7 h, the reaction mixture was poured into water and extracted with dichloromethane. The organic layer was collected and dried over anhydrous MgSO4. The mixture was concentrated under reduced pressure. Resulting solid was stirred in a mixture of dichloromethane (18.00 mL) and methanol (6.00 mL), followed by potassium carbonate (0.23 g, 1.69 mmol). After stirring for 4 h, white solid was precipitated from the solution. The crude product was washed alternately with dichloromethane and water to obtain **IM1**(131 mg, 48%). ^{1}H NMR (400 MHz, DMSO-*d*$_6$) δ 8.65 (s, 1H), 8.53 (s, 1H), 7.95 (d, J = 8.6 Hz, 1H), 6.62 (d, J =

8.6 Hz, 1H), 6.20 (s, 2H), 5.58 (d, J = 9.0 Hz, 1H), 4.17 (t, J = 8.9 Hz, 1H), 3.87 (m, 2H), 3.41-3.51 (m, 7H).

1.2. Determination of LOD

The linear fitting curve of absorption or emission of **PPAB-Gal** and addition concentration of BA was provide the slope over a fixed linear range. The LOD value was calculated by the equation of LOD = 3σ/K, where σ is the standard error of the intercept of the fitted straight line.

1.3. Smartphone-Based on-site and Semi-Quantitative Analysis of BAs

In this experiment, a smartphone-based chromatic analysis application (Color Finder) by Apple Store was adopted and applied under daylight and 365 nm irradiation to semi-quantify the color change of **PPAB-Gal**. BA was incubated with a **PPAB-Gal**-based TLC plates for a desired time. Next, the corresponding photographs were taken with a smartphone (iPhone 13) under daylight and 365 nm irradiation. The colors of the images were subsequently split directly into the RGB channel intensity by a third-party application on a smartphone (Color Finder). The limit of quantitation detection (LQD) of **PPAB-Gal** toward BAs was calculated as LOQ = 10δ/k based on the quantitative detection of SEM by the smartphone APP, where δ is the standard deviation of blank measurement and k is the slope of the calibration curve.

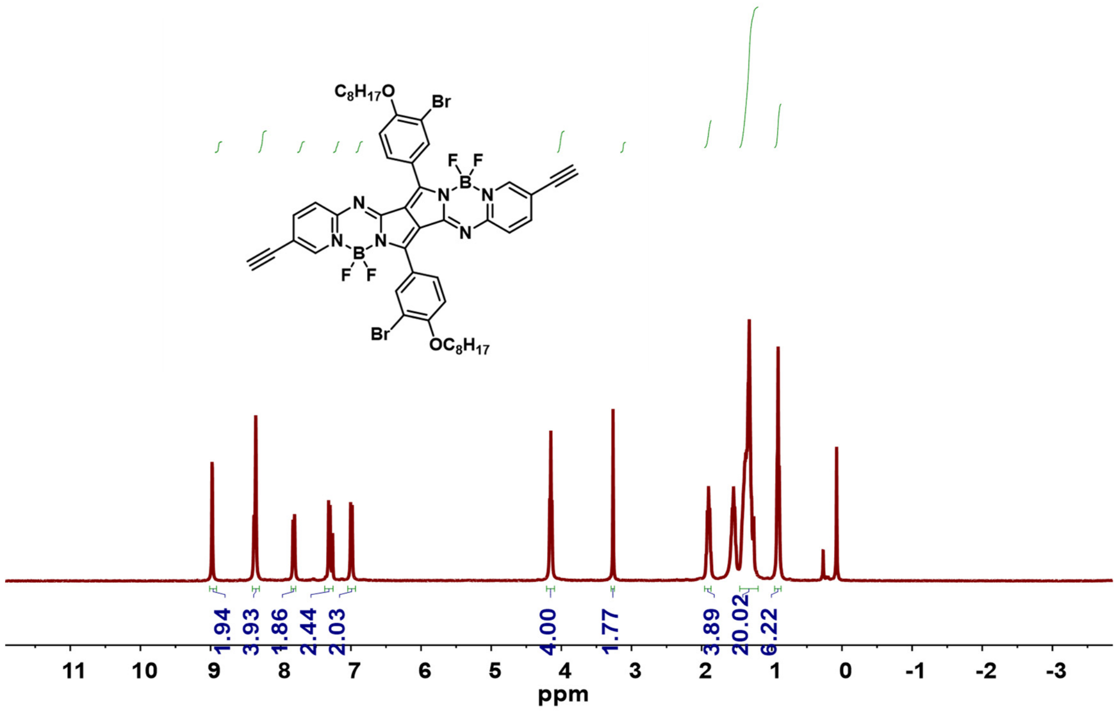

Figure S1. ^{1}H NMR spectrum of **PPAB-2** in $CDCl_3$.

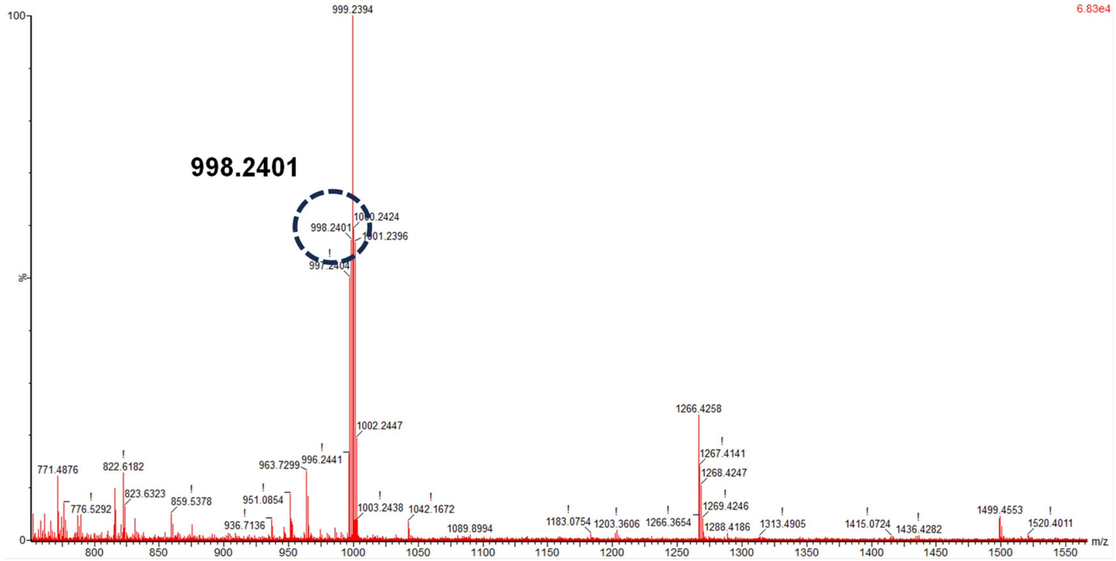

Figure S2. The TOF spectrum of **PPAB-2**.

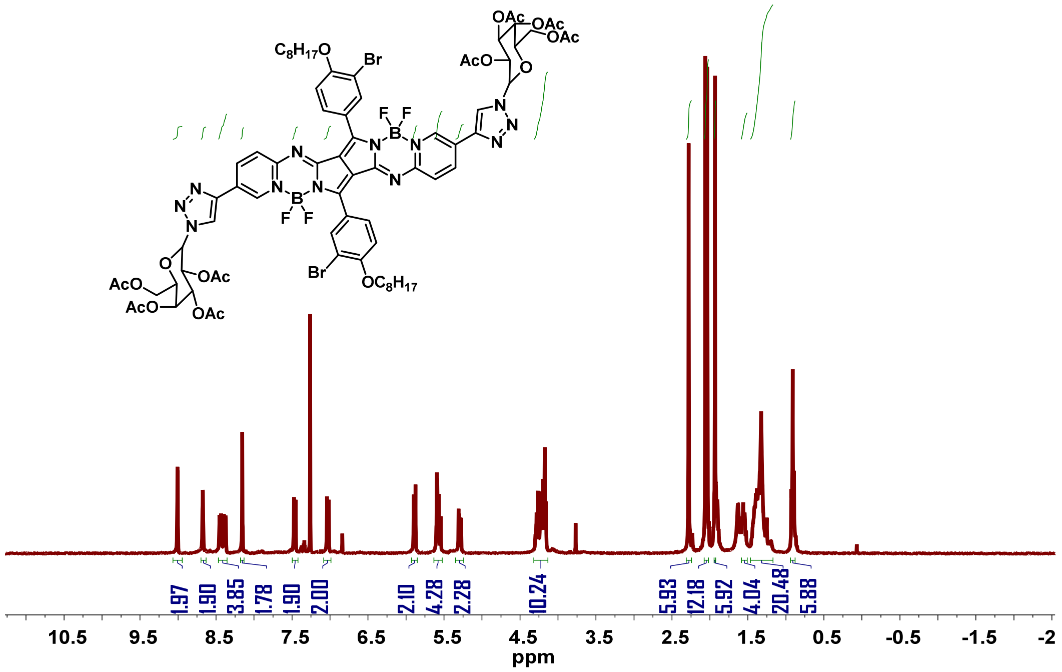

Figure S3. ^{1}H NMR spectrum of **PPAB-OAc** in $CDCl_3$.

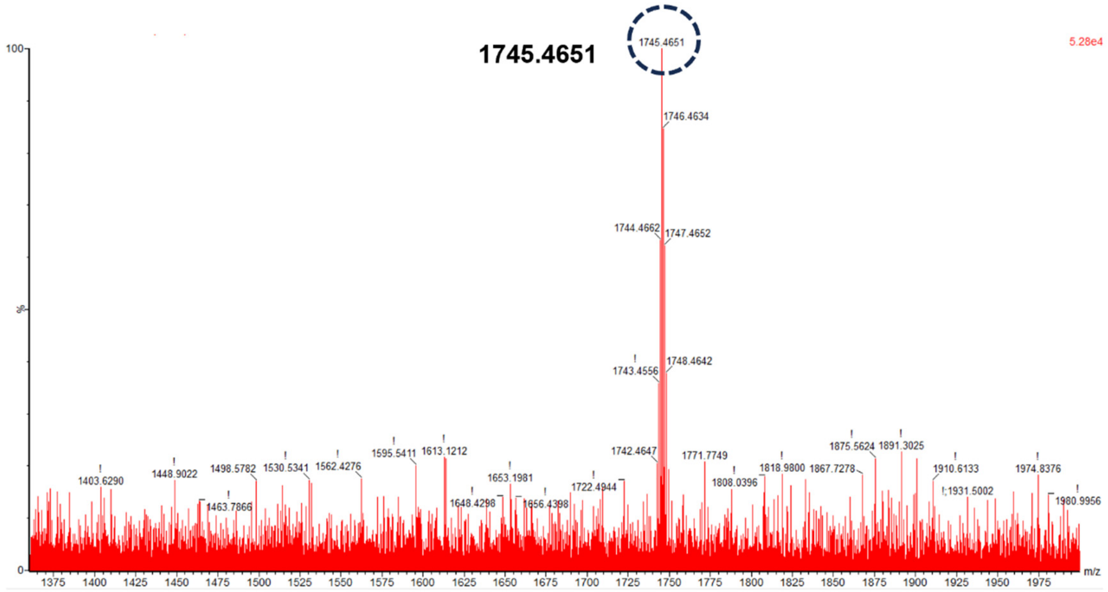

Figure S4. The TOF spectrum of **PPAB-OAc**.

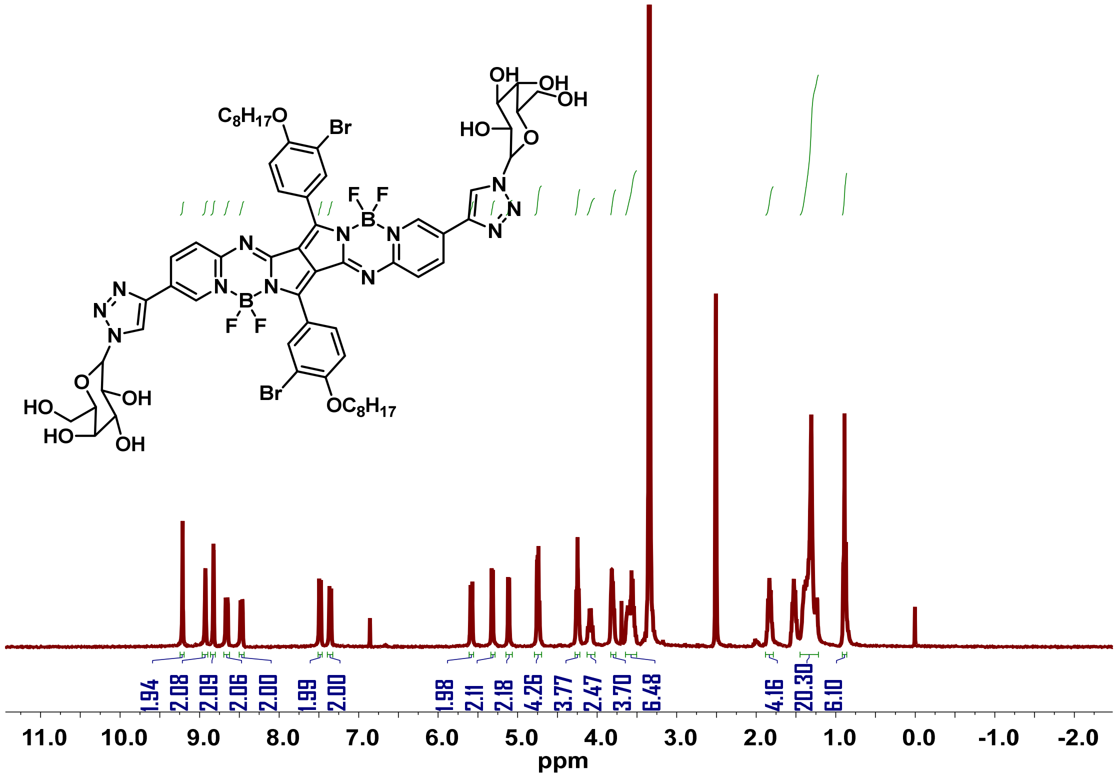

Figure S5. ^{1}H NMR spectrum of **PPAB-Gal** in DMSO-*d*$_6$.

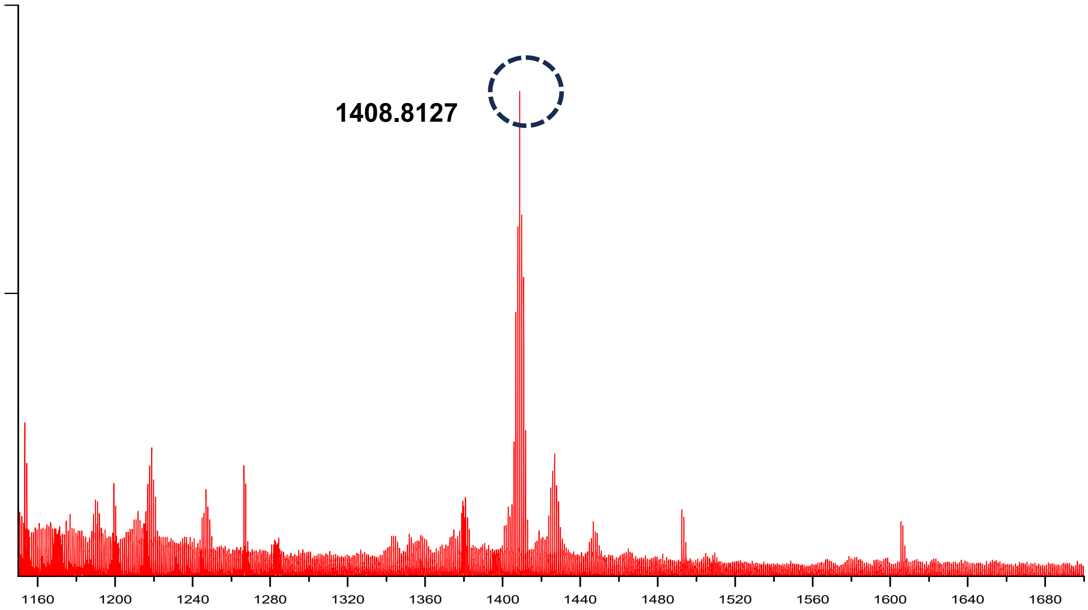

Figure S6. The TOF spectrum of **PPAB-Gal**.

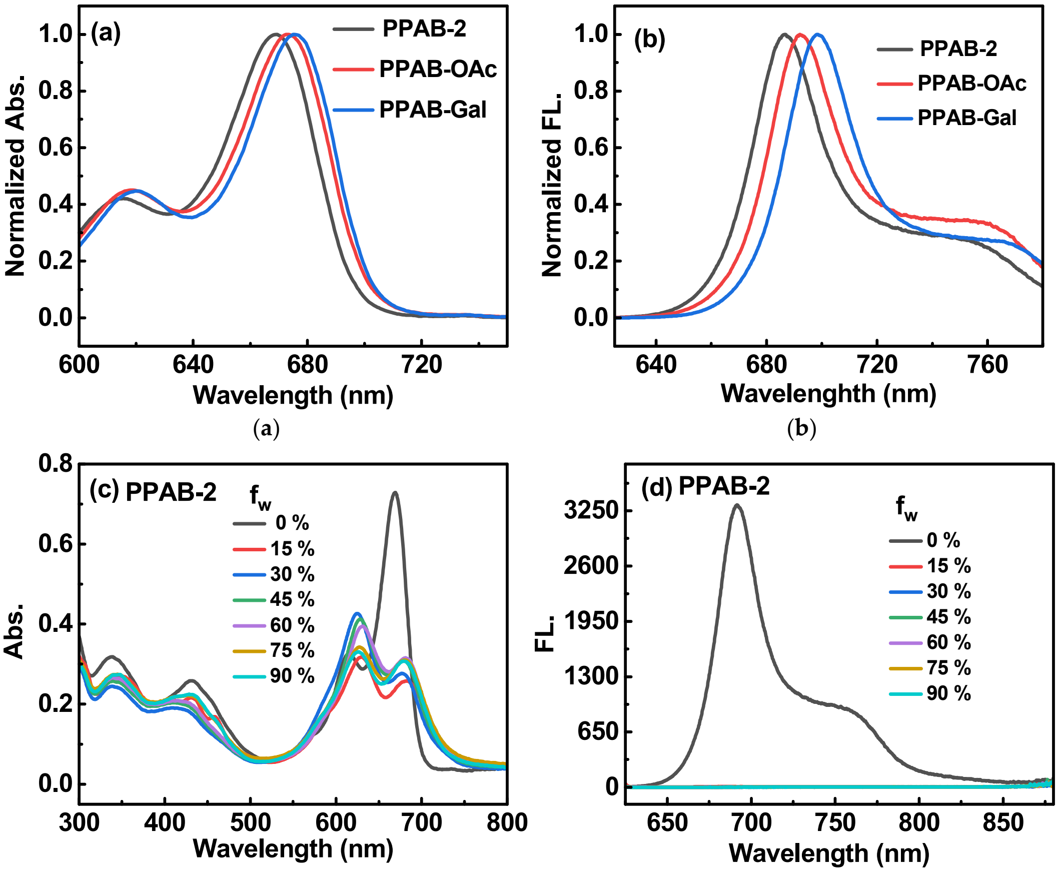

(**a**) (**b**)

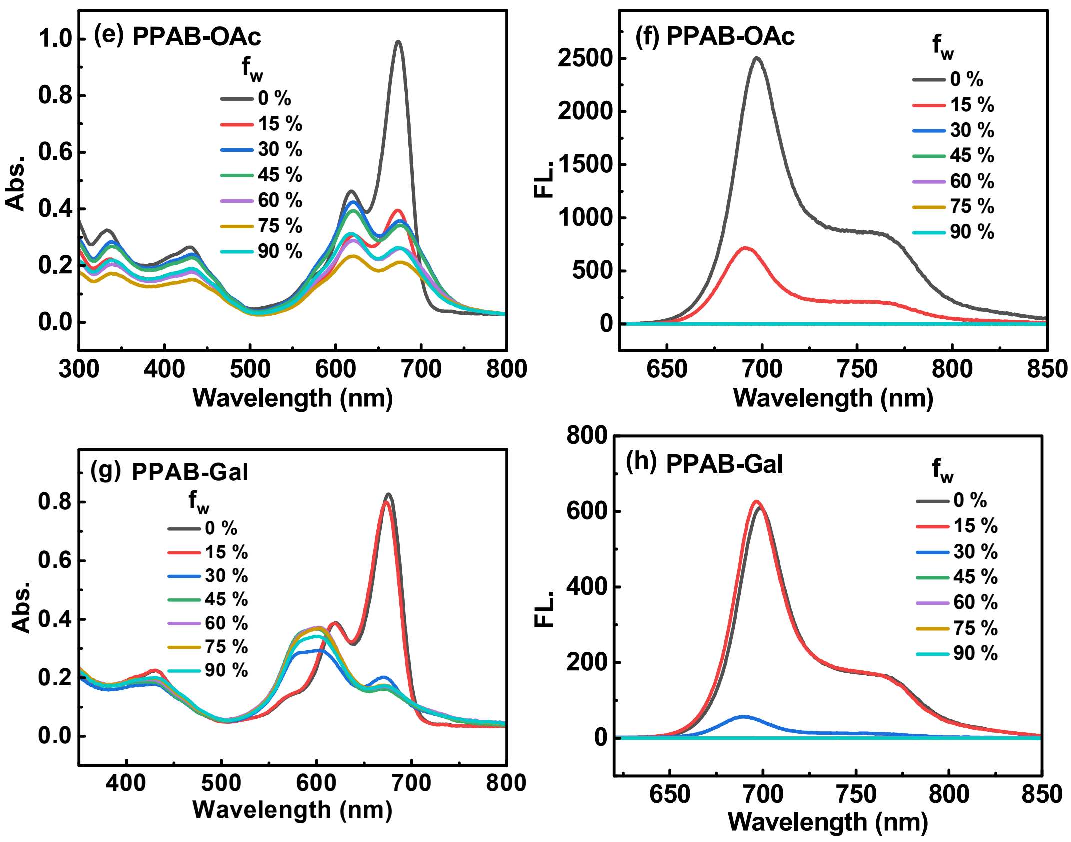

Figure S7. (a, b) Normalized absorption and emission spectra of **PPAB-2**, **PPAB-OAc**, **PPAB-Gal** in CH_2Cl_2. Absorption and emission spectra of **PPAB-2** (c, d), **PPAB-OAc** (e, f), **PPAB-Gal** (g, h) in DMSO/H_2O mixture with different fraction of H_2O (f_w).

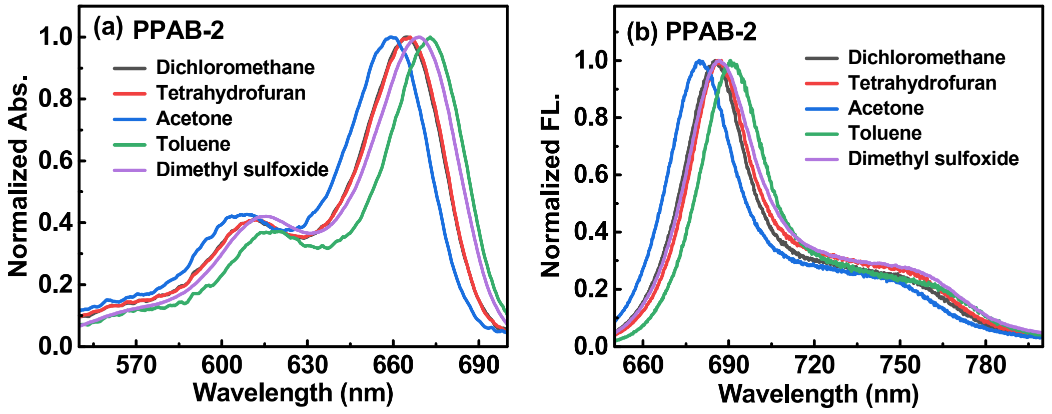

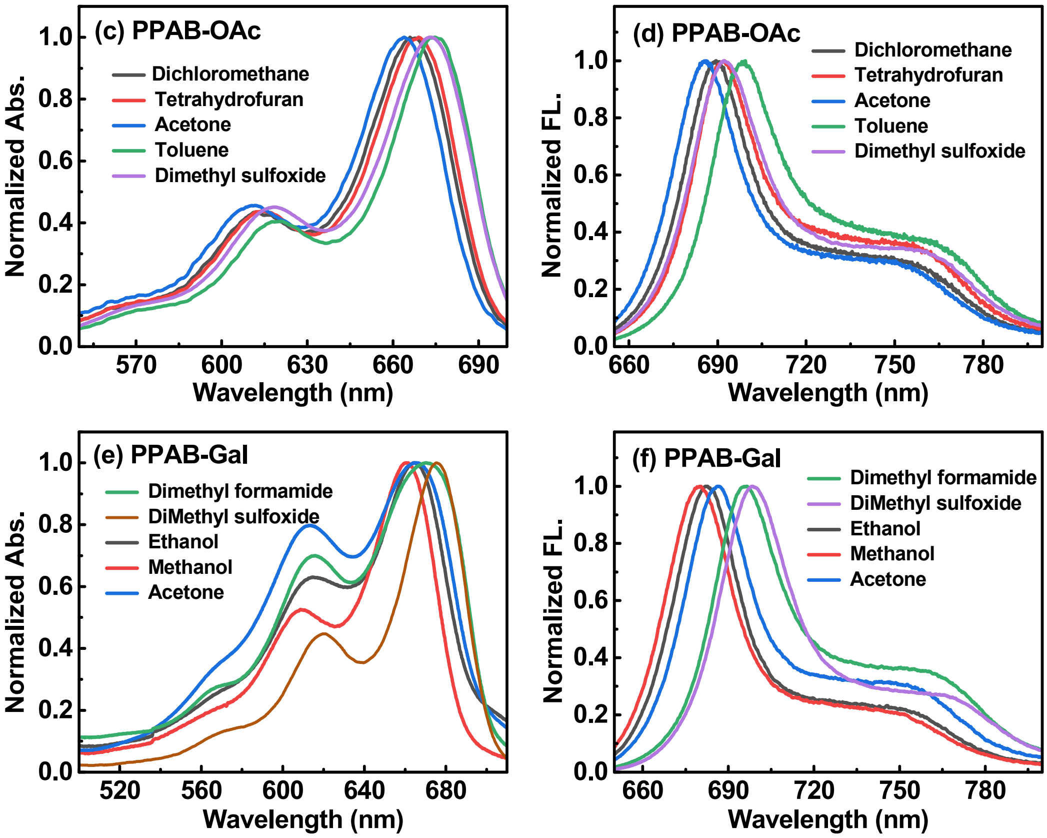

Figure S8. Normalized absorption and emission spectra of **PPAB-2** (a, b), **PPAB-OAc** (c, d), and **PPAB-Gal** (e, f) in different solvents.

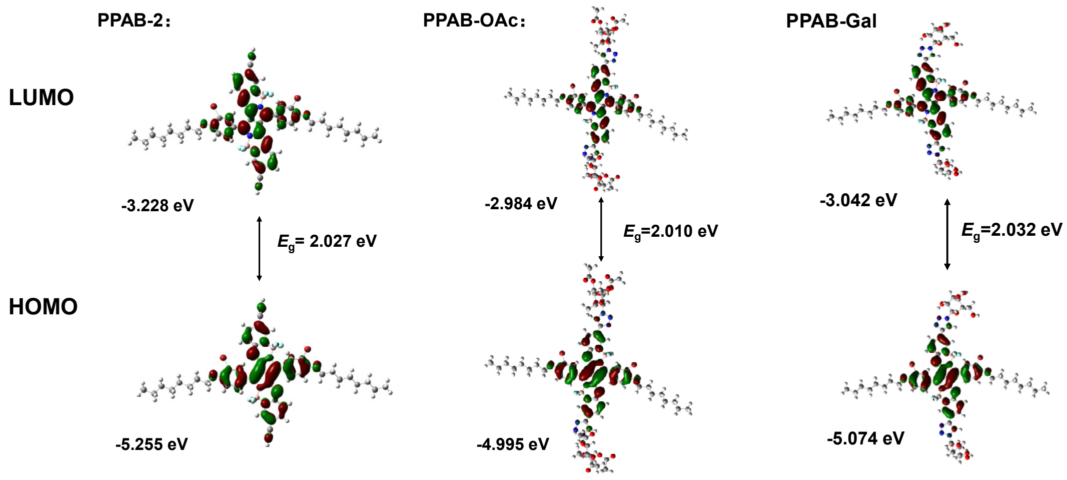

Figure S9. The DFT results of **PPAB-2**, **PPAB-OAc** and **PPAB-Gal**.

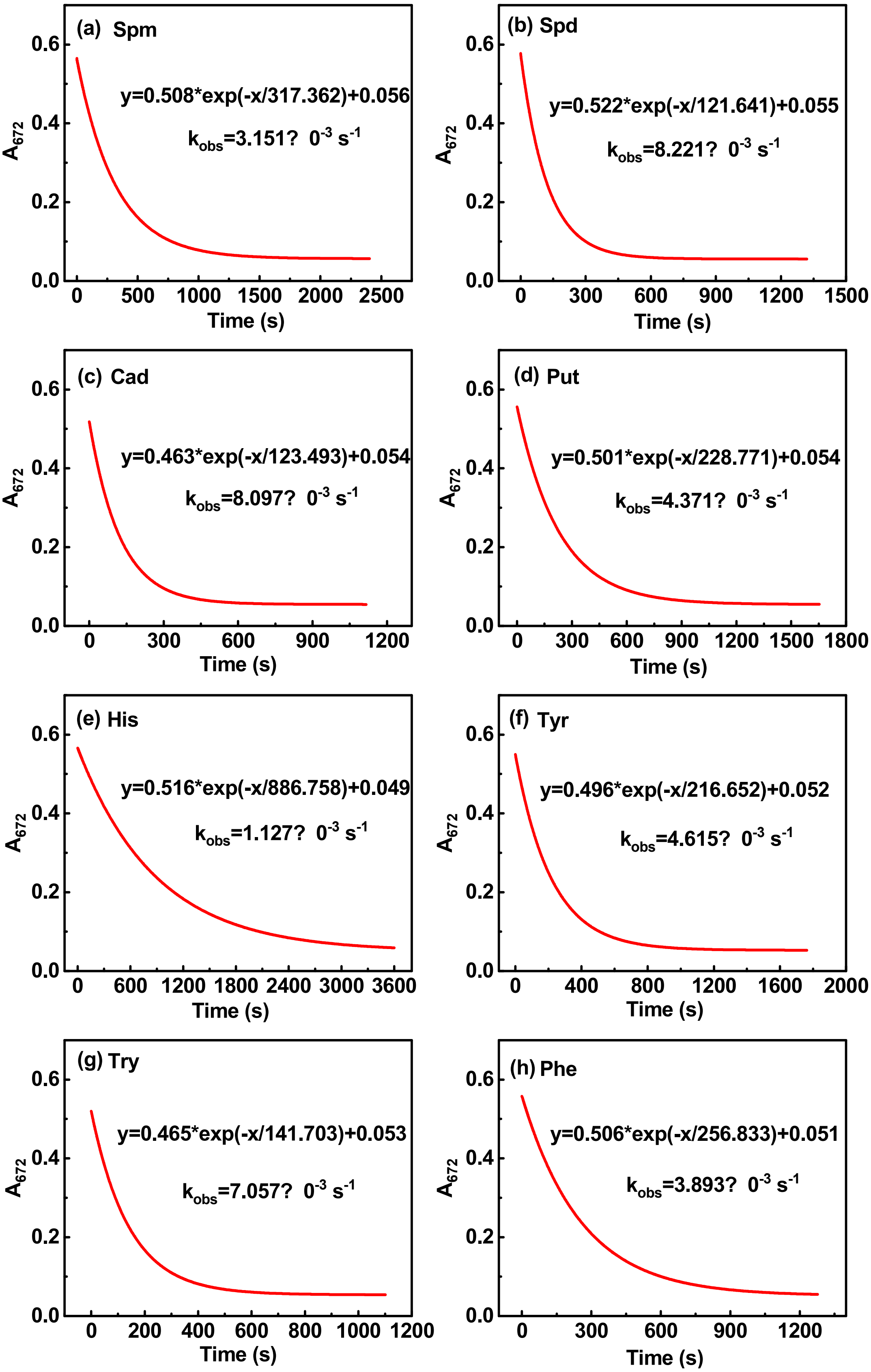

Figure S10. The reaction kinetic **PPAB-Gal** (10 μM) in presence of (a) Spm, (b) Spd, (c) Cad, (d) Put (e) His, (f) Try, (g) Tyr, (h) Phe (200 μM) in DMSO/H_2O (4/1, v/v).

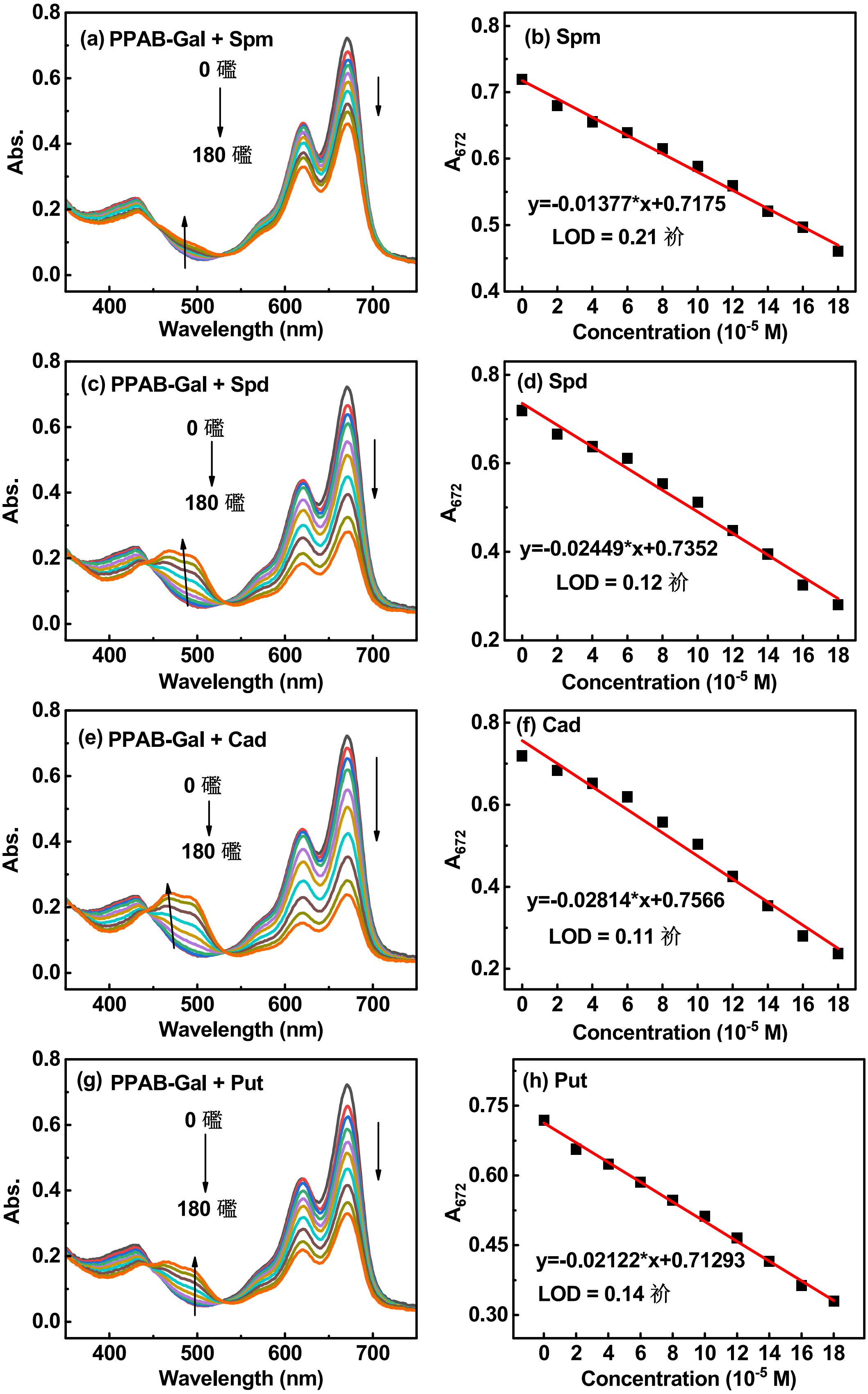
(a) PPAB-Gal + Spm
0 礚
180 礚
Abs.
Wavelength (nm)
(b) Spm
y=-0.01377*x+0.7175
LOD = 0.21 祄
A_{672}
Concentration (10^{-5} M)
(c) PPAB-Gal + Spd
0 礚
180 礚
Abs.
Wavelength (nm)
(d) Spd
y=-0.02449*x+0.7352
LOD = 0.12 祄
A_{672}
Concentration (10^{-5} M)
(e) PPAB-Gal + Cad
0 礚
180 礚
Abs.
Wavelength (nm)
(f) Cad
y=-0.02814*x+0.7566
LOD = 0.11 祄
A_{672}
Concentration (10^{-5} M)
(g) PPAB-Gal + Put
0 礚
180 礚
Abs.
Wavelength (nm)
(h) Put
y=-0.02122*x+0.71293
LOD = 0.14 祄
A_{672}
Concentration (10^{-5} M)

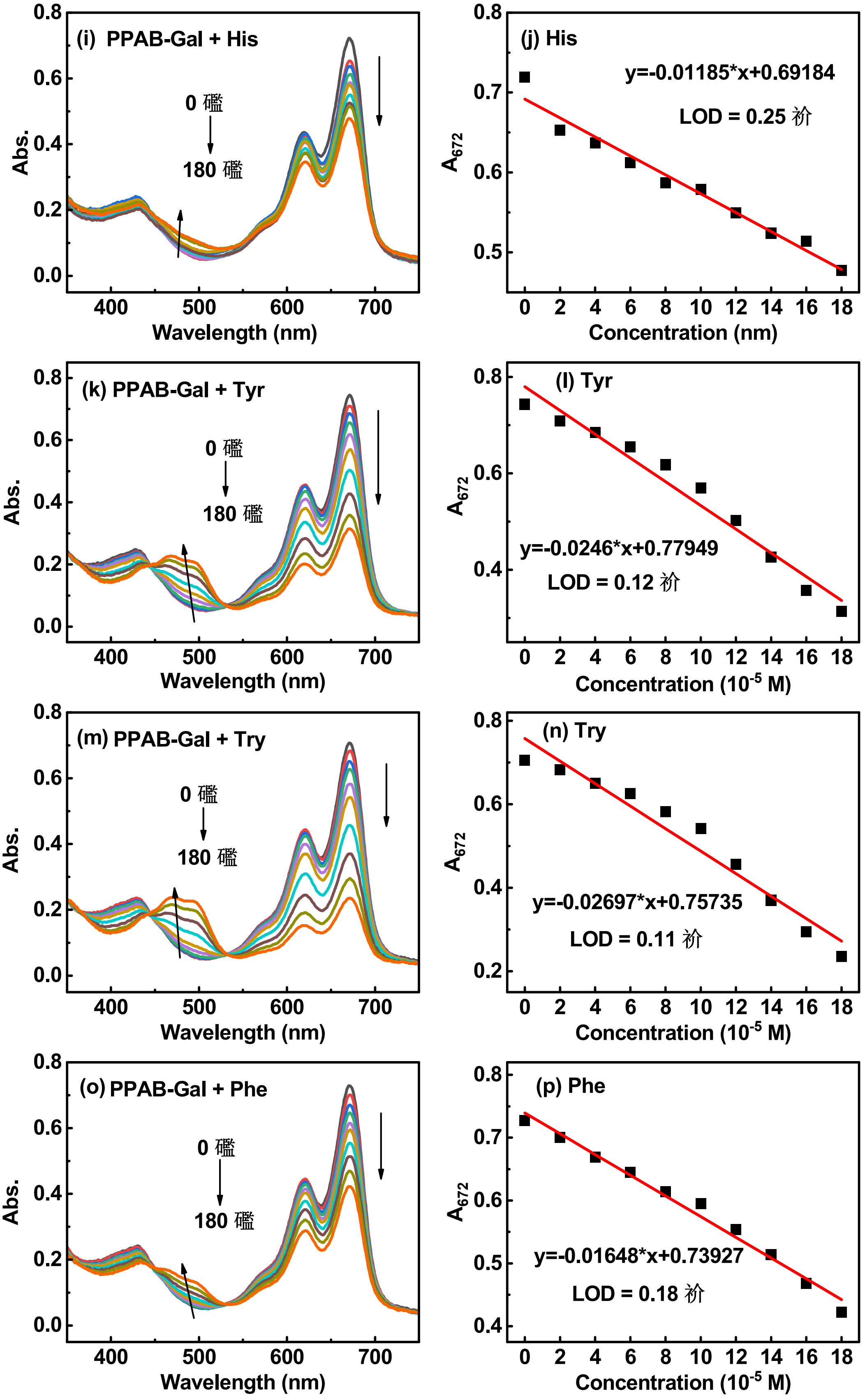
(i) PPAB-Gal + His
0 礛
180 礛
Abs.
Wavelength (nm)
(j) His
y=-0.01185*x+0.69184
LOD = 0.25 衸
A_{672}
Concentration (nm)
(k) PPAB-Gal + Tyr
0 礛
180 礛
Abs.
Wavelength (nm)
(l) Tyr
y=-0.0246*x+0.77949
LOD = 0.12 衸
A_{672}
Concentration (10^{-5} M)
(m) PPAB-Gal + Try
0 礛
180 礛
Abs.
Wavelength (nm)
(n) Try
y=-0.02697*x+0.75735
LOD = 0.11 衸
A_{672}
Concentration (10^{-5} M)
(o) PPAB-Gal + Phe
0 礛
180 礛
Abs.
Wavelength (nm)
(p) Phe
y=-0.01648*x+0.73927
LOD = 0.18 衸
A_{672}
Concentration (10^{-5} M)

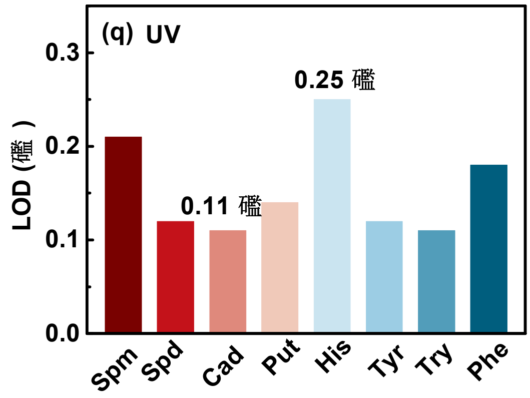

Figure S11. (a-p) The concentration-dependent UV-vis spectra and linear plot of **PPAB-Gal** in presence of 8 BAs in DMSO/H_2O (4/1, v/v). (q) The LOD values of 8 BAs.

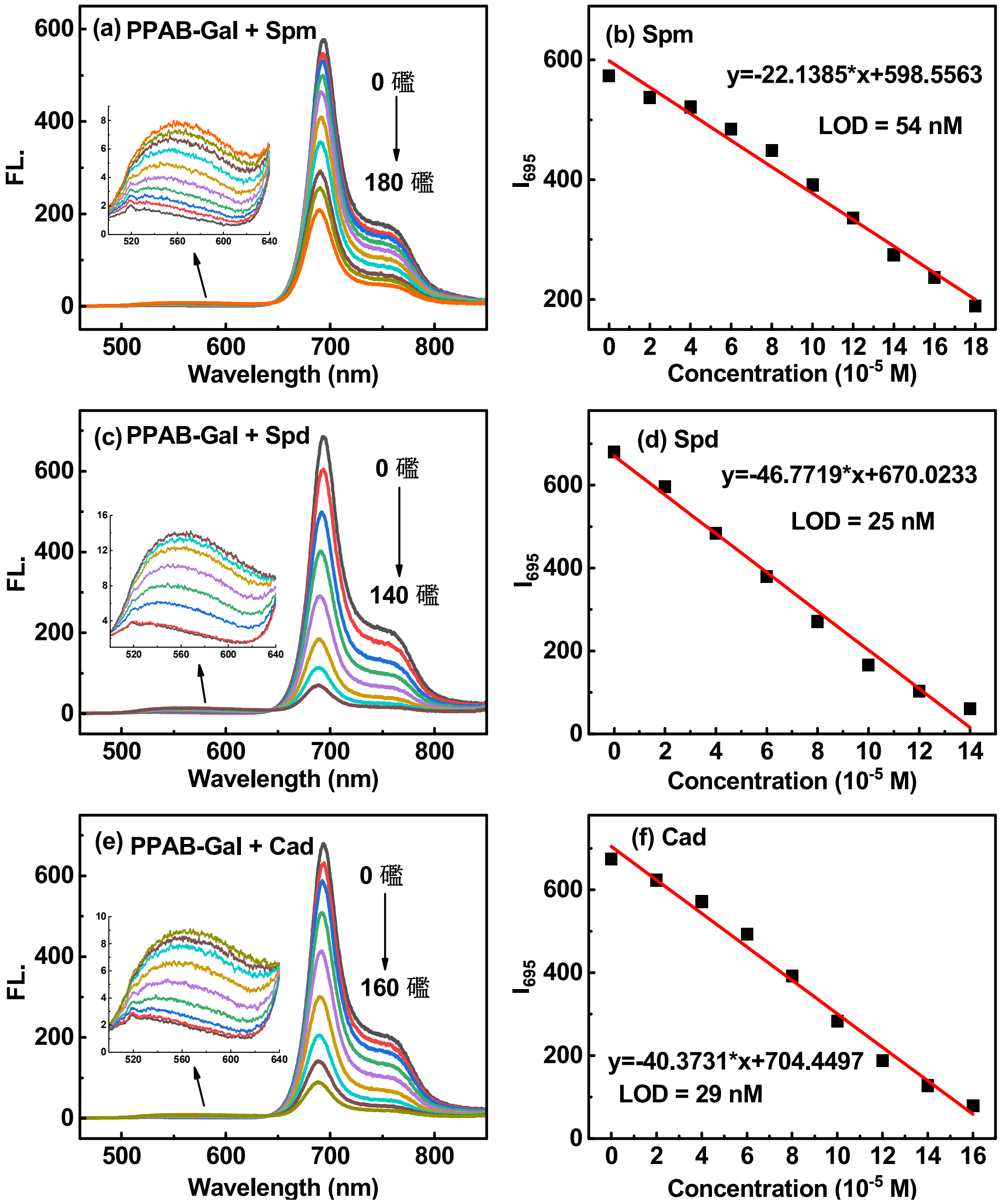

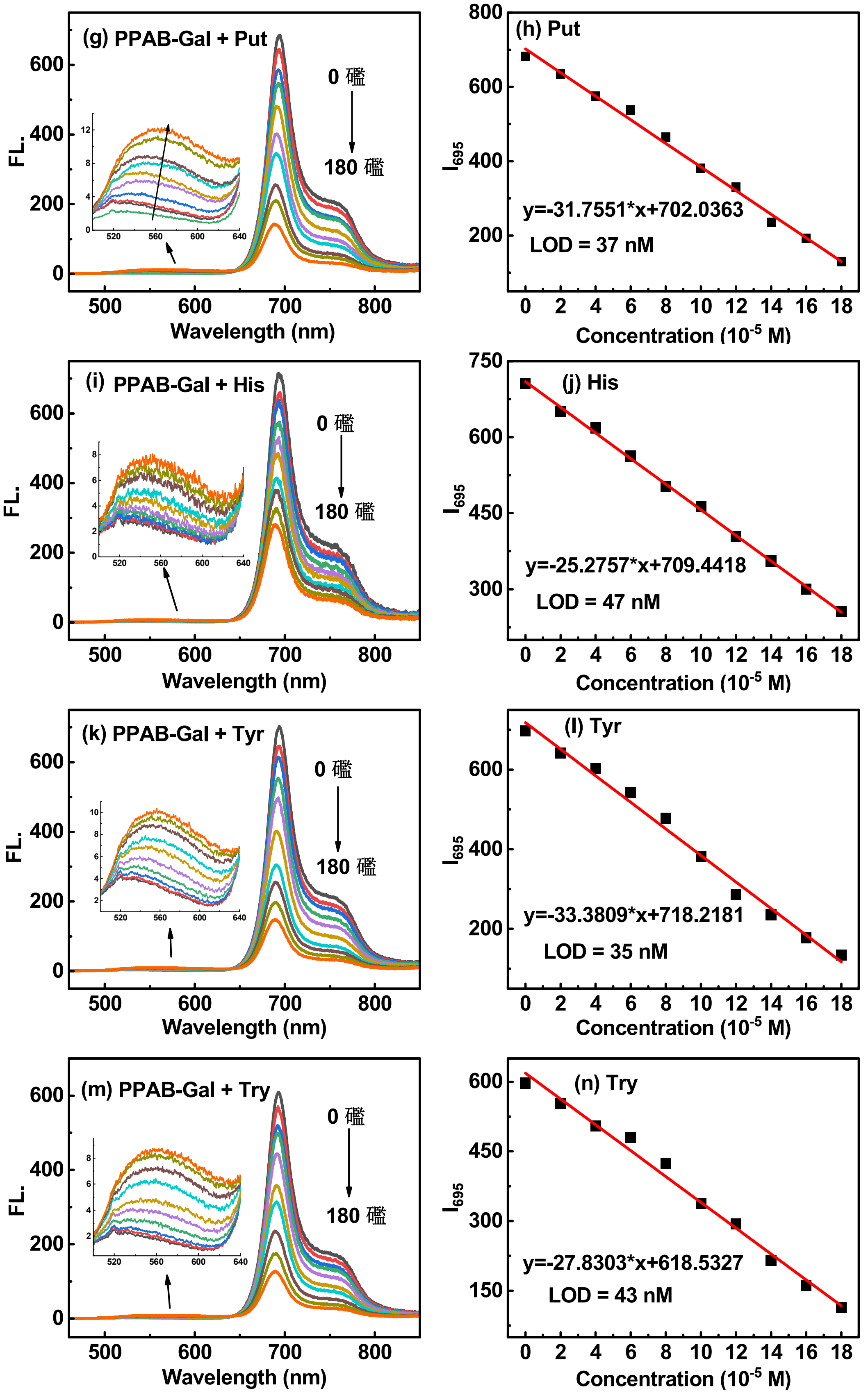

(g) PPAB-Gal + Put
0 礚
180 礚
FL.
Wavelength (nm)
(h) Put
I695
y=-31.7551*x+702.0363
LOD = 37 nM
Concentration (10-5 M)
(i) PPAB-Gal + His
0 礚
180 礚
FL.
Wavelength (nm)
(j) His
I695
y=-25.2757*x+709.4418
LOD = 47 nM
Concentration (10-5 M)
(k) PPAB-Gal + Tyr
0 礚
180 礚
FL.
Wavelength (nm)
(l) Tyr
I695
y=-33.3809*x+718.2181
LOD = 35 nM
Concentration (10-5 M)
(m) PPAB-Gal + Try
0 礚
180 礚
FL.
Wavelength (nm)
(n) Try
I695
y=-27.8303*x+618.5327
LOD = 43 nM
Concentration (10-5 M)

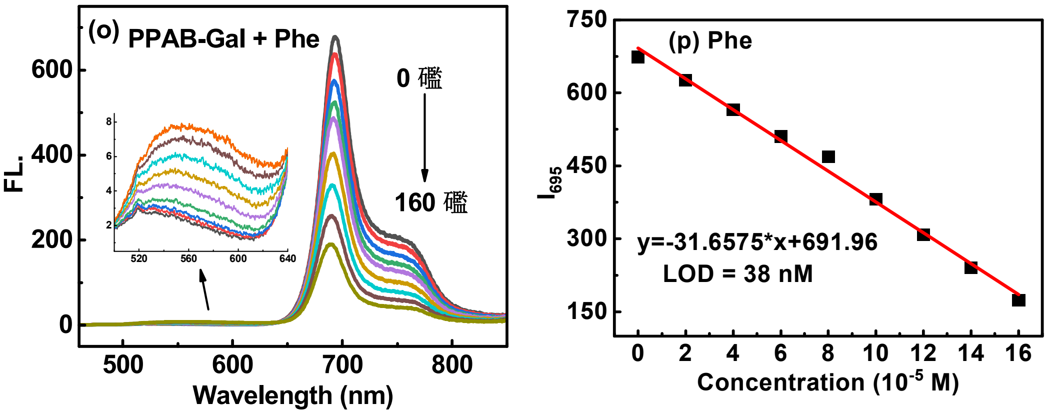

Figure S12. (a-p) The concentration-dependent emission spectra and linear plot of **PPAB-Gal** in presence of 8 BAs in DMSO/H_2O (4/1, v/v).

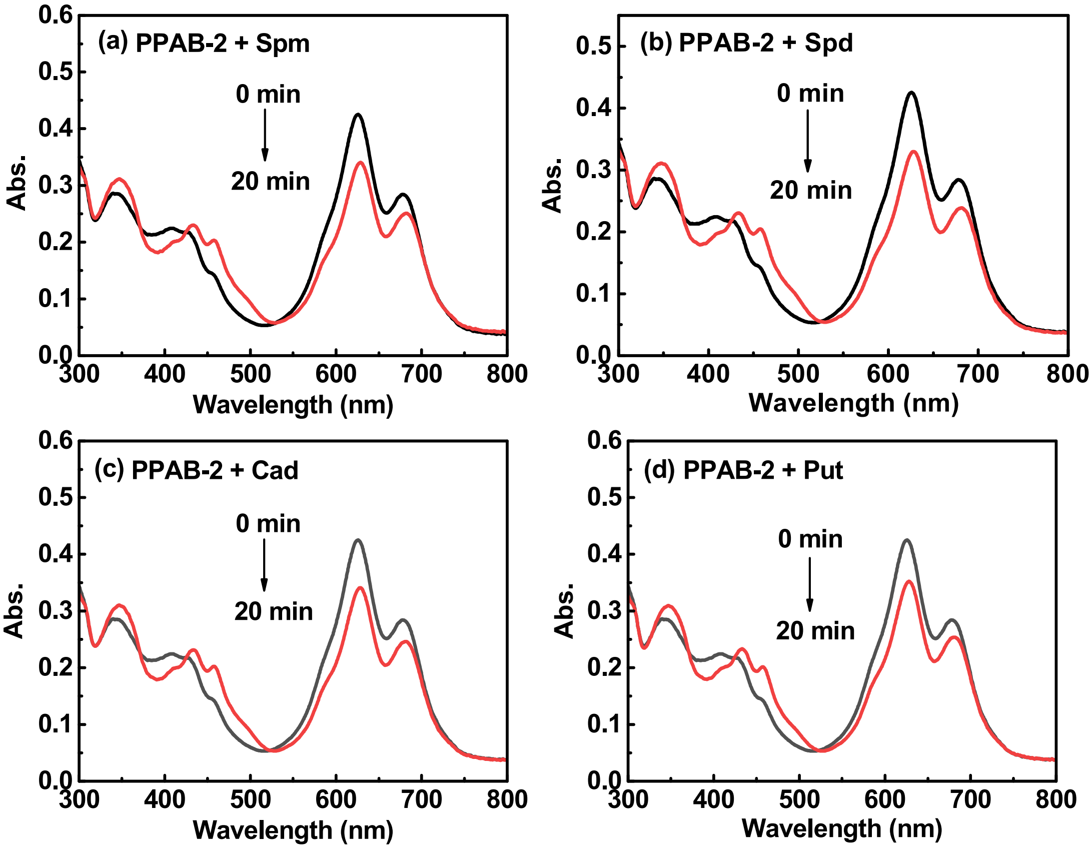

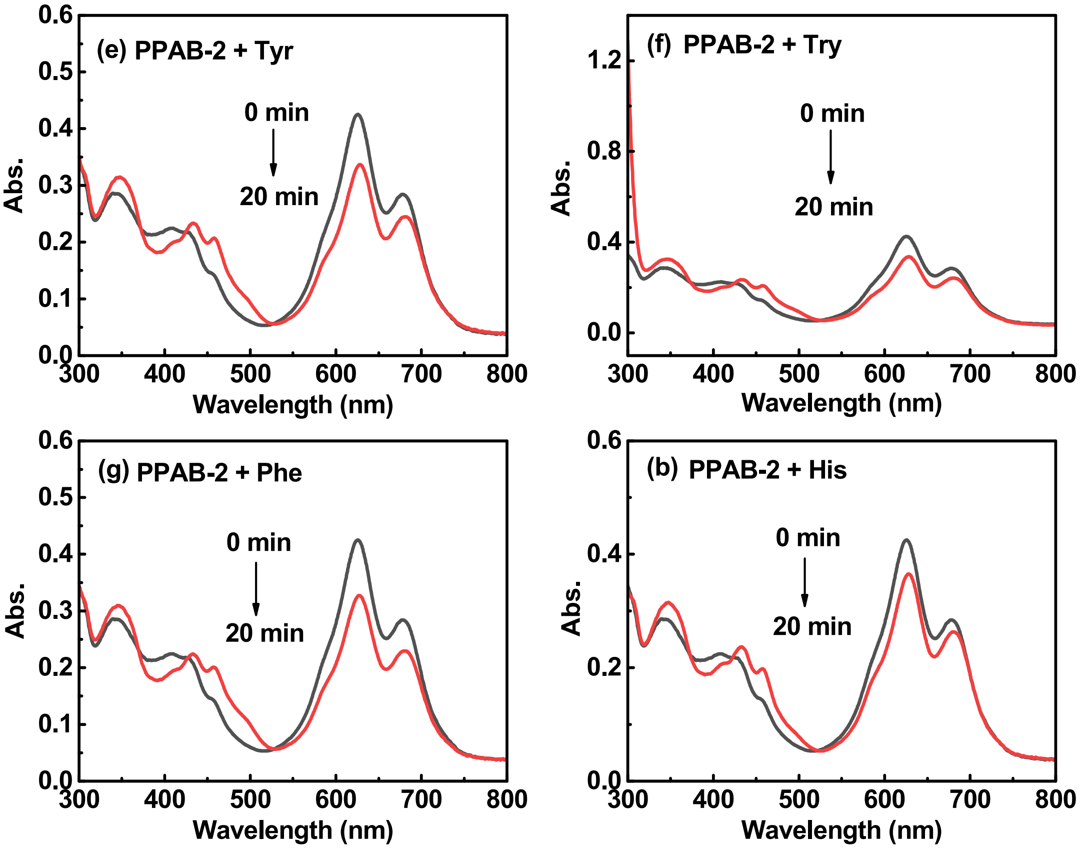

Figure S13. Time-dependent of absorption spectra of **PPAB-2** (10 μM) in presence of (a) Spm, (b) Spd, (c) Cad, (d) Put, (e) Try, (f) Tyr, (g) Phe and (h)His (200 μM) in DMSO/H_2O (4/1, v/v).

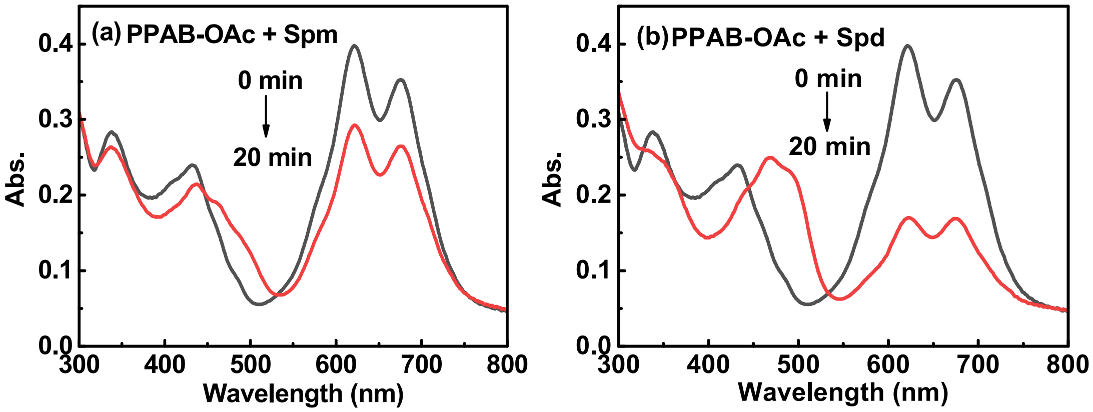

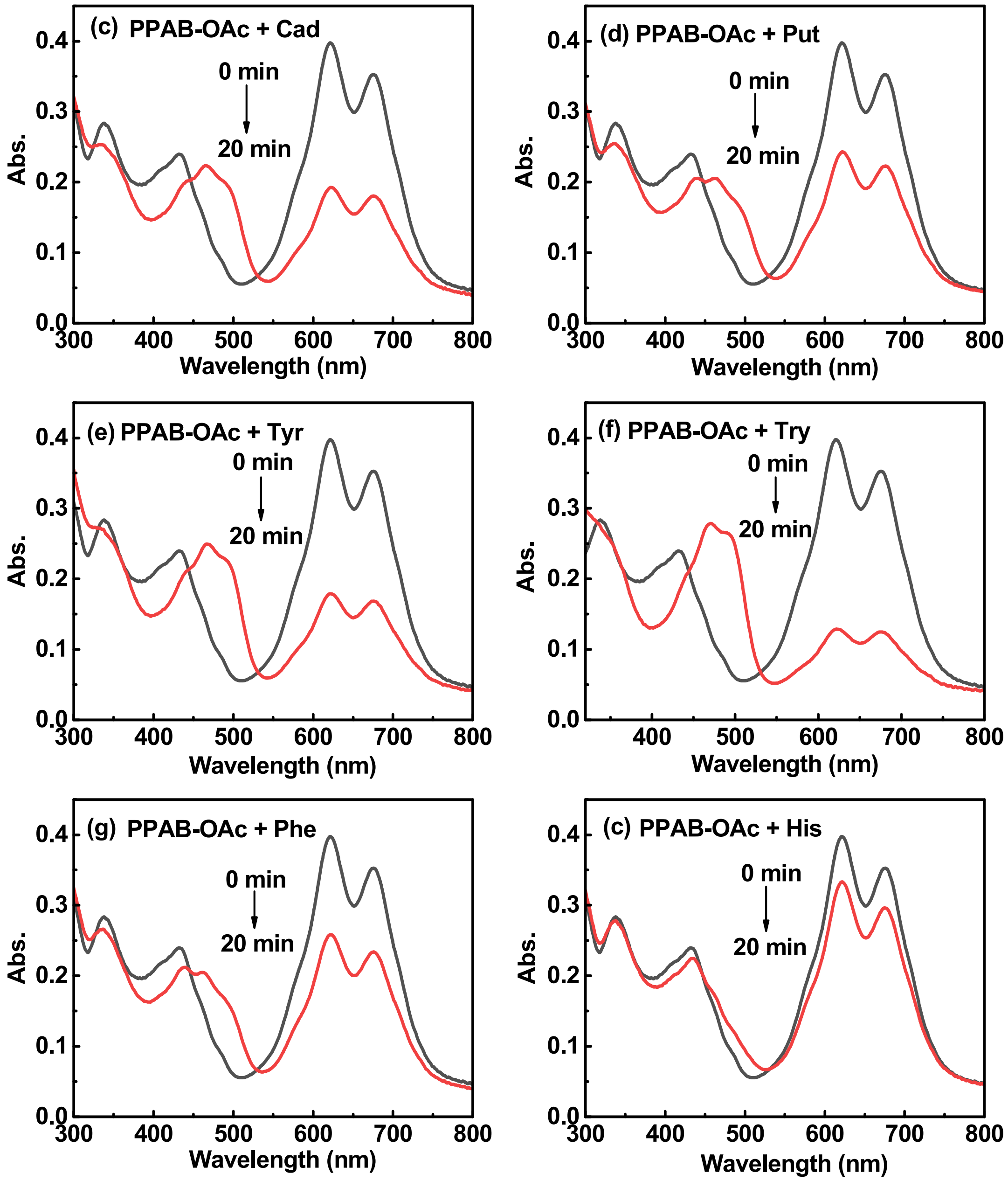

Figure S14. Time-dependent of UV-vis absorption spectra of **PPAB-OAc** (10 μM) in presence of (a) Spm, (b) Spd, (c) Cad, (d) Put (e) Try, (f) Tyr, (g) Phe, and (h)His (200 μM) in DMSO/H2O (4/1, v/v).

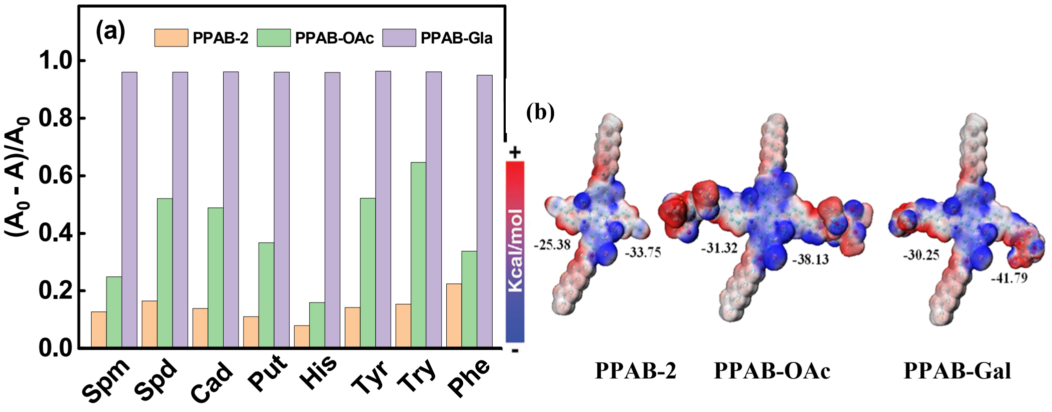

Figure S15 (a) The reaction kinetics curves of **PPAB-Gal** towards 8 BAs. (b) The electrostatic surface potential (ESP) maps of **PPAB-2**, **PPAB-OAc**, and **PPAB-Gal**.

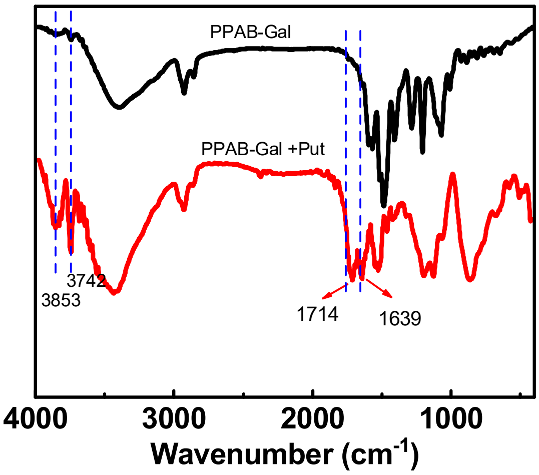

Figure S16. The FT-IR spectra of **PPAB-Gal** in presence of Put.

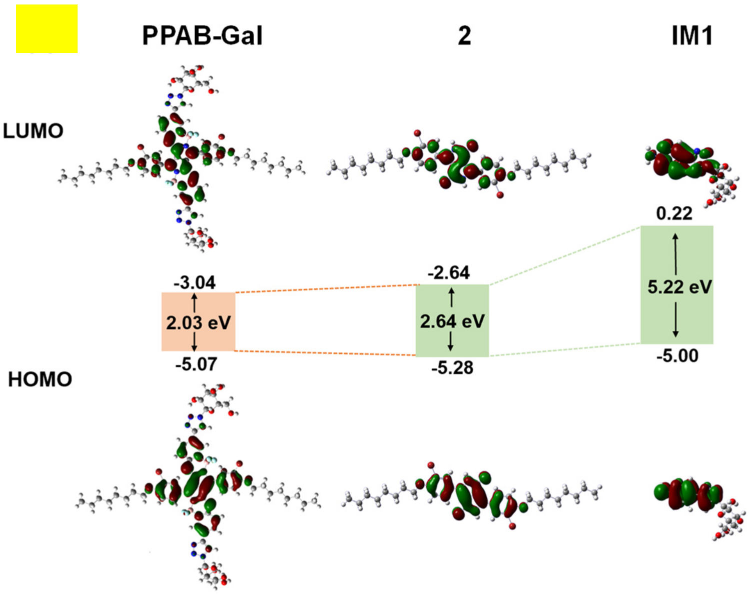

Figure S17. The DFT results of **PPAB-Gal** and possible products (compounds **2** and **IM1**).

(a) PPAB-Gla +Phe

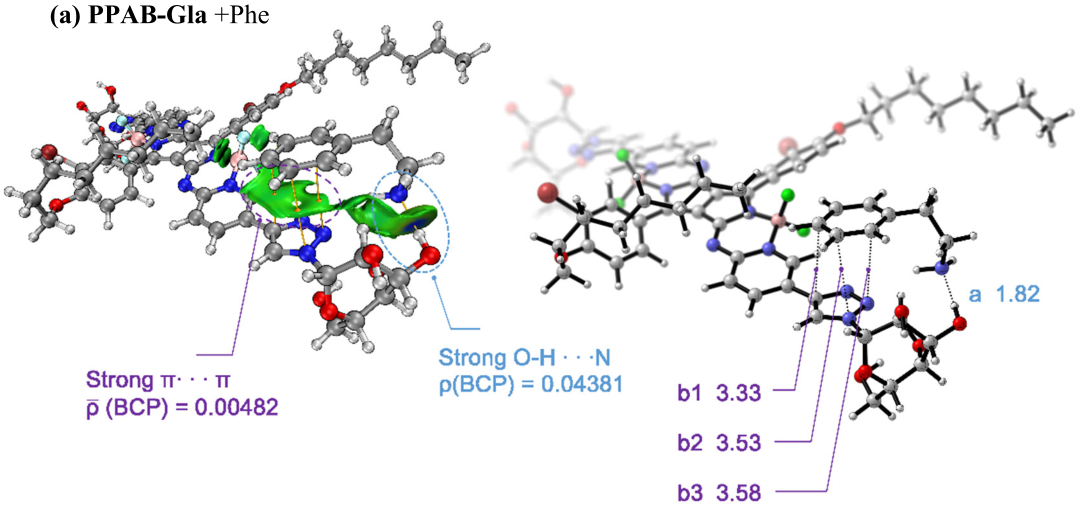

(b) PPAB-Gla + Try

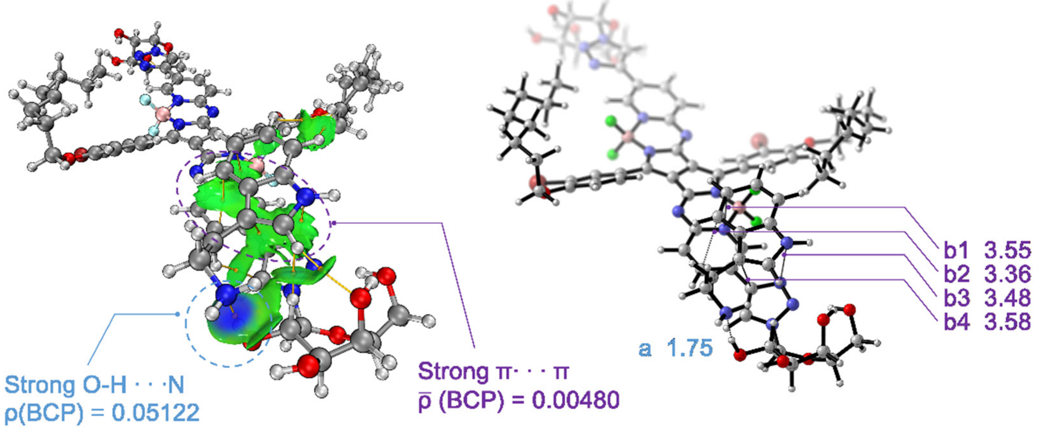

(c) PPAB-Gla + His

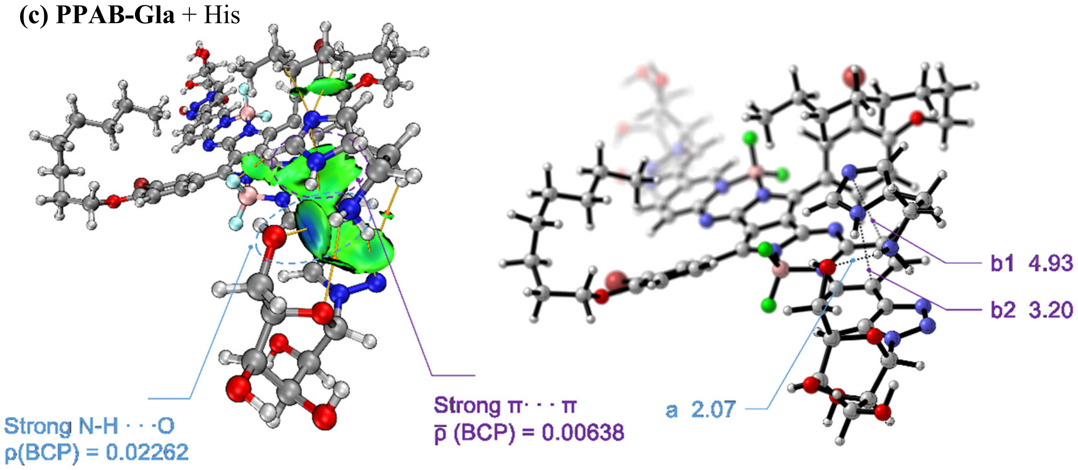

Figure S18. Optimized molecular structure (gaseous state) of **PPAB-Gal** and Phe, Try and His with selected interatomic distances (Å).

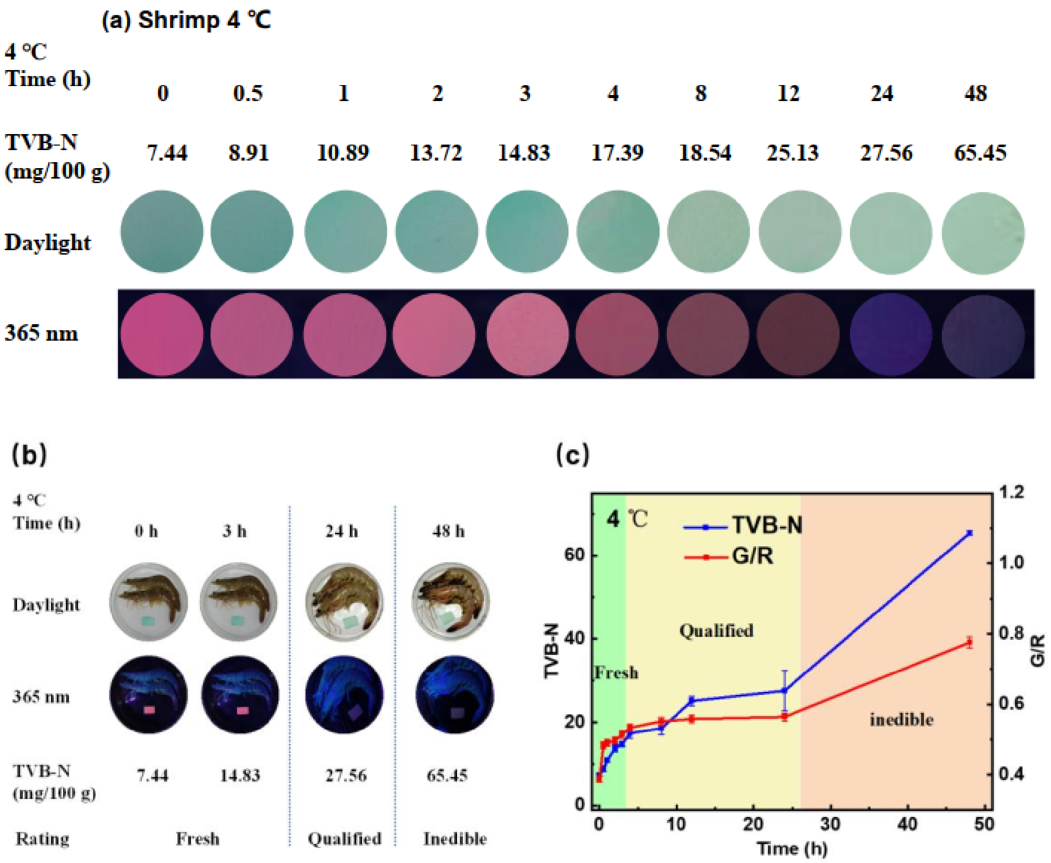

Figure S19. (a, b) The color change and emission of **PPAB-Gal** -loaded TLC plate for shrimp at 4 °C. (c) The grades of shrimp freshness evaluation by measuring TVB-N value using a smartphone sensing platform based on the color of **PPAB-Gal** -loaded TLC plate under UV light at 4 °C. .

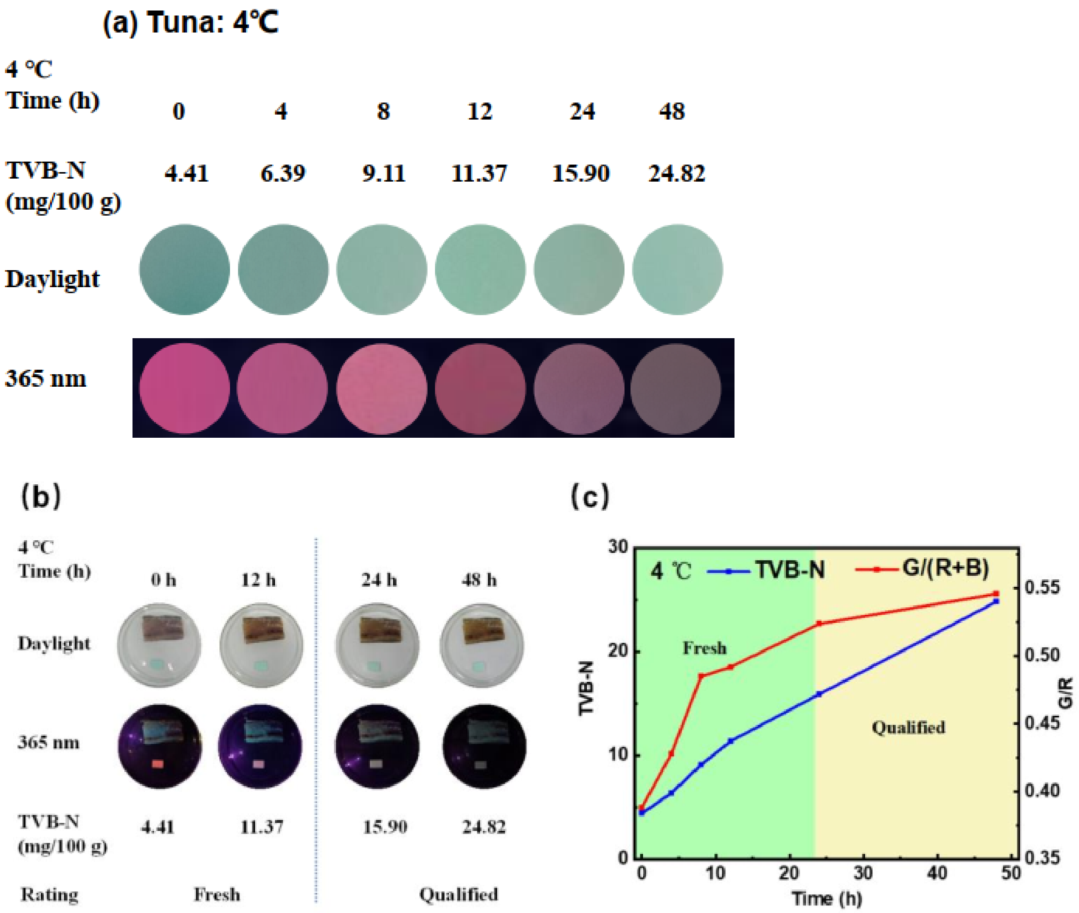

Figure S20. (a, b) The photos of color change and emission of **PPAB-Gal** -loaded TLC plate for tuna at 4 °C. (c) The grades of tuna freshness evaluation by measuring TVB-N value using a smartphone sensing platform based on the color of **PPAB-Gal** -loaded TLC plate under UV light at 4 °C. .

Table S1. The photophysical data of **PPAB-2**, **PPAB-OAc** and **PPAB-Gal** in different solvents.

Compound	Solvent	$\lambda_{max,abs}$ (nm)	λ_{em} (nm)	Stokes shift (nm)	ε ($M^{-1}\cdot cm^{-1}$)
**PPAB-2**	CH_2Cl_2	665	685	20	1.31×10^5
	THF	666	686	20	1.25×10^5
	Acetone	659	680	21	5.35×10^4
	Toluene	673	691	18	1.28×10^5
	DMSO	669	687	18	7.28×10^4
**PPAB-OAc**	CH_2Cl_2	667	689	22	1.35×10^5
	THF	669	692	23	1.33×10^5
	Acetone	664	686	22	7.75×10^4
	Toluene	675	699	24	1.30×10^5
	DMSO	673	692	19	9.91×10^4
**PPAB-Gal**	Ethanol	664	682	18	/
	Methanol	661	680	19	/
	Acetone	665	687	22	/
	DMF	671	696	25	7.48×10^4
	DMSO	676	699	23	8.28×10^4

Table S2. The comparison of fluorescent probes for BAs detection in food.

Chemical structure	Sensing mechanism	Amines	Remarks	Ref.
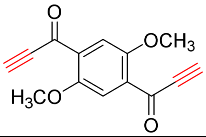	Amino-alkyne click polymerization	Put	High sensitivity (30 s), LOD = 3.19 × 10^{-7}M	RSC Adv., 2022, 12, 26630–26638
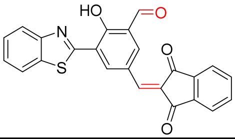	Formation two Schiff bases	Cad and Put	fast response (<15 min), LOD = 70 nM to Cad	Food Chem., 2024, 436, 137769
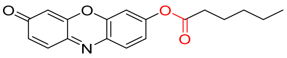	Nucleophilic substitution reaction	Cad and Put	Rapid response (< 8 min), LOD = 0.47 mM, detection of BAs in living cells and zebrafish	RSC Adv., 2022, 12, 33870–33875
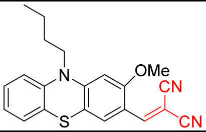	Reversed-Knoevenagel condensation& aldehyde– amine condensation	Cad	LOD = 46 nM, fast response (< 15 s)	Analyst, 2022, 147, 923–931
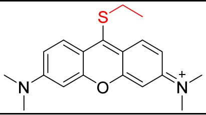	Nucleophilic substitution reaction	Tyr, Try, Cad, Spd, Spm, His, aniline, propylamine	Rapid response, LOD = 30 nM	Anal. Chim. Acta., 2024, 1285, 342025
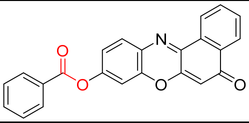	Nucleophilic Substitution reaction	Methylamine	Rapid response (30 s), good selectivity, LOD = 17 nM	Spectrochim. Acta Part A: 2023, 302, 123004
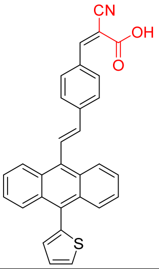	Complex and proton exchange	Put, Cad and Spm	Naked-eye detection of the BA vapor	J. Mater. Chem. B, 2024, 12, 2746–2760
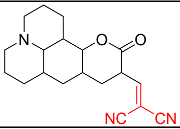	Aza-Michael addition	Cad	Visual color change, fast response, LOD = 51 nM	Food Chem., 2022, 394, 133489
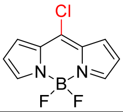	Sequential nucleophilic addition-elimination reaction	Cad, Put, Spm and Spd	LOD = 5.1 nM	Dyes Pigm., 2023, 220, 111771
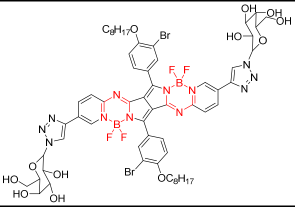	Chromophore reaction	Cad, Put, Spm, Spd, His, Try, Tyr, Phe (8 BAs)	Rapid response(< 2 min), responsive to 8 BAs, LOD below to 25 nM, $\Delta\lambda_{ab}$ = 130 nm, $\Delta\lambda_{em}$ = 150 nm	This work

Table S3. Summary of different BAs detection methods.

Type	Sensing mechanism	Advantages	Disadvantages
High-performance liquid chromatography (HPLC)	Due to the lack of suitable chromophores for most biogenic amines, derivatization with reagents such as dansyl chloride (Dns-Cl) and o-phthalaldehyde (OPA) is necessary before the detection of fluorescence or ultraviolet.	High accuracy, sensitivity, and selectivity	The derivatization process is time-consuming and laborious. They rely highly on expensive equipment, greatly limiting their applicability in field settings.
Colorimetric mode	The reaction of biogenic amines with other substances to produce noticeable color changes to qualitatively and/or quantitatively detect BAs.	Simple, low cost, on site, real-time, non-destructive detection	It cannot be used beyond a certain concentration with low sensitivity, semi-quantitative and easy to cross reaction.
Fluorescence mode	The interaction between binding units (or receptors) and biogenic amines, which would change the fluorescence signal through different communication mechanisms	Satisfactory sensitivity, fast response, high-throughput, non-destructive	High background signals, single signal, easy to cross reaction.
Chemiluminescence mode (direct luminescence, catalyzed luminescence, induced luminescence, etc)	A chemical reaction is generated in an electronically excited state and returns to its ground state under emission of light.	Assembly like quickness, easy operation, simple procedure, low detection limit.	Different light resource (normally in the visible and/or near-infrared regions), only response to few analytes.
This work	Noncovalent interactions (π–π stacking, and hydrogen bond, *etc*)-assisted chromophore reaction	Simultaneously detect 8 aliphatic and aromatic BAs, distinctly dual signals LOD (~ 25 nM) and fast response time (< 2 min), qualitative and smartphone-assisted sensing platform for visual detecting freshness of tuna and shrimp.	Improve the water solubility of probe and sensitivity.
